# Yap Is a Nutrient Sensor Sensitive to the Amino Acid L-Isoleucine and Regulates the Expression of Ctgf in Cardiomyocytes

**DOI:** 10.3390/biom14101299

**Published:** 2024-10-14

**Authors:** Victoria L. Nelson, Ashley L. Eadie, Lester Perez, Malav Madhu, Mathew Platt, Angella Mercer, Thomas Pulinilkunnil, Petra Kienesberger, Jeremy A. Simpson, Keith R. Brunt

**Affiliations:** 1 Department of Pharmacology, Dalhousie University, Halifax, NS B3H 4R2, Canada; 2 Dalhousie Medicine New Brunswick, Faculty of Medicine, Dalhousie University, Saint John, NB E2L 4L5, Canada; 3 IMPART Investigator Team Canada, Saint John, NB E2L 4L5, Canada; 4 Department of Human Health and Nutritional Sciences, University of Guelph, Guelph, ON N1G 2W1, Canada; 5 Department of Biochemistry and Molecular Biology, Dalhousie University, Halifax, NS B3H 4R2, Canada

**Keywords:** heart failure, hippo signalling, mitochondria, mechanotransduction

## Abstract

Myocardial infarction and reperfusion constitute a complex injury consisting of many distinct molecular stress patterns that influence cardiomyocyte survival and adaptation. Cell signalling, which is essential to cardiac development, also presents potential disease-modifying opportunities to recover and limit myocardial injury or maladaptive remodelling. Here, we hypothesized that Yap signalling could be sensitive to one or more molecular stress patterns associated with early acute ischemia. We found that Yap, and not Taz, expression patterns differed in a post-myocardial infarct compared to a peri-infarct region of rat hearts post-myocardial infarction, suggesting cell specificity that would be challenging to resolve for causation *in vivo*. Using H9c2 ventricular myotubes *in vitro* as a model, Yap levels were determined to be more sensitive to nutrient deprivation than other stress patterns typified by ischemia within the first hour of stress. Moreover, this is mediated by amino acid availability, predominantly L-isoleucine, and influences the expression of *connective tissue growth factor* (*Ctgf*)—a major determinant of myocardial adaptation after injury. These findings present novel opportunities for future therapeutic development and risk assessment for myocardial injury and adaptation.

## 1. Introduction

Despite extensive research, myocardial infarction (MI) continues to be a leading cause of morbidity and mortality worldwide [1]. Thus, identifying mechanisms of cardiomyocyte adaptation after MI is imperative for the discovery of new therapeutic strategies. MI can be characterized as either reduced blood flow or its cessation in a coronary artery, causing ischemia—the deprivation of oxygen/nutrients and the accumulation of metabolic waste causing oxidative stress, cell death, and inflammation [2]. The origins of ischemia are diverse and include coronary vasospasm; the build-up of atherosclerosis coronary plaques, as well as their rupture/erosion; and cardiac microvascular dysfunction and rarefaction (the loss or eradication of small blood vessels and their collateralization). Occlusive ischemia of the myocardium can be lethal if blood flow is not rapidly restored [3]. Even with reperfusion, there is an associated injury that can irreversibly compromise cardiac function and then progress to heart failure or arrhythmia morbidity. Current therapeutic interventions (e.g., using angiotensin II receptor blockers, angiotensin-converting-enzyme inhibitors, beta-blockers, and anti-platelet/coagulants) reduce cardiac workload or increase coronary perfusion [2,4], yet therapeutics for deliberately promoting adaptive cardiac remodelling post-MI are still required to improve patient outcomes.

Various cells coordinate mechanisms to maintain the heart’s contractile capacity and structural integrity to keep up with the body’s metabolic demands. Many molecular mechanisms are perturbed by cardiac ischemia and resolve in a distinct timeline for each cell type, with pathophysiological consequences. Cell survival mechanisms are some of the first adaptations to ischemia, allowing resistance to the necrosis and/or apoptosis of the cardiomyocytes post-MI [5]. Compensating for cell loss is critical to preserve mechanical and contractile functions to maintain cardiac output, with fibrotic stabilization and then hypertrophy of the surviving cardiomyocytes being central to short-term adaptation in the infarct and peri-infarct regions of a ventricular free wall under pressure/strain [6]. Cardiac hypertrophy limits wall stress and maintains function by increasing myofilament number and efficiency, without cell proliferation [7]. Initially beneficial, wall chamber shape can elongate with hypertrophy, becoming eccentric and generating less contractile force; such maladaptive remodelling can ultimately lead to heart failure [6]. What ischemia triggers early in cardiac molecular signalling after MI can, therefore, reveal potential early activation or repression patterns that might be useful in terms of informing interventions.

Pathways regulating cardiac development likely present an opportunity to improve cardiomyocyte adaptation post-MI. Among these pathways, Yap—a key component to the Hippo signalling pathway—plays a direct role in the size regulation of the heart during development [8]. Yap signalling is regulated by several extrinsic and intrinsic stimuli that can initiate the cascade and are generally considered part of mechanical cell signaling, which would be relevant to the late remodelling of a dilating ventricle [9]. The extent of Yap signalling’s early sensitivity to acute ischemia is unknown. Canonically, when Yap is unphosphorylated, it translocates to the nucleus, where it binds to a transcription factor(s), predominantly TEAD, to initiate transcription [10]. When Yap is phosphorylated, it is retained in the cytoplasm and, in some cases, leads to proteasomal degradation [11,12]. Yap signalling is involved in the tissue repair process in other organs (i.e., intestines, liver) and is essential in maintaining tissue homeostasis and promoting regeneration after injury [13,14]. There is evidence that active Yap reduced cardiac scar formation and ultimately improved cardiac function post-MI via *Yap* overexpression [15,16] or the deletion of *Sav*1 in mice [17]. Nevertheless, the cell types and/or underlying triggers induced by ischemia remain unclear. Unlike other organs, the heart has limited regenerative potential due to an inability to efficiently make new cardiomyocytes [18]. Therefore, exploring the role of cardiac Yap signalling in response to MI and the underlying mechanisms of ischemia affecting Yap could improve existing cardiac remodelling post-MI or provide new possibilities for promoting the adaptive responses of cardiomyocytes.

In this study, we sought to characterize the role of Yap signalling in response to early ischemia stressors and drivers of cardiac injury. By modelling the component stressors related to ischemia, we identified that Yap signalling is sensitive to nutrient deprivation, regulated in part by L-isoleucine. Furthermore, the expression of a Yap-associated *Ctgf* gene was significantly increased after this stimulus was induced in the first hour of nutrient deprivation. These results establish that Yap is metabolically sensitive and that Yap-regulated signalling in the infarcted myocardium is immediate, potentially affecting longer-term remodelling and adaptation, presenting a novel target for therapy or risk assessment.

## 2. Methods

### 2.1. In Silico Analyses

The Gene Expression Omnibus (GEO), created by the National Center for Biotechnology Information (NCBI), is a resource for high-throughput datasets that follow the NCBI’s guidelines for Minimum Information About a Microarray Experiment (MIAME) [19]. Microarray data sets related to MI were found using the search terms “myocardial infarchtion” and “rat” in the GEO database. Gene expression was investigated in the left ventricle of male Wistar rats in which either small, moderate, or large Mis were induced via proximal left-coronary-artery ligation (Series Accession GDS4907). Gene expression was compared to that in sham animals (which were subject to the same surgery without ligation). The data presented constitute relative mRNA (log2-transformed) [20].

### 2.2. Animal Care

Animal care and all experimental procedures were carried out per Canadian Council on Animal Care guidelines and approved by the University of Guelph’s Animal Care Committee. Animals were provided food and water *ad libitum* and maintained on a light/dark (12:12 h) cycle.

### 2.3. Pre-Clinical Models

Acute myocardial infarction was induced via permanent ligation of the left anterior descending (LAD) coronary artery in C57BL/6 adult male mice (8 weeks of age). Animals were anesthetized (2%:100% for isofluorane/O_2_), intubated, and ventilated using the Harvard Apparatus at 200 breaths/minute. Para-sternal thoracotomy was performed under sterile surgical conditions. Next, a 7-0 Surgipro^TM^ II polypropylene suture (Covidien; Dublin, Ireland) was used to ligate the LAD below the atrioventricular border, which was confirmed via blanching of the wall below the ligation. The same operation was performed for Sham animals, but no ligation was carried out. After surgery, animals were checked twice daily for any complications. AMI animals and shams were humanely slain and their organs were harvested at 7 and 28 days post-injury to assess expression in the remodelling phase post-MI.

### 2.4. Tissue Collection and Preparation

Hearts were excised and immediately flash-frozen and stored at −80 °C until use. Samples were ground using mortar and pestle while in liquid nitrogen. A total of 10–15 mg of the sample was homogenized for 30 s in 120 μL NP-40-based lysis buffer (1% NP-40) and then placed on ice for 30 min. Fresh lysis buffer was made by adding a 1:100 phosphatase inhibitor (PHI) cocktail (524628, EMD Millipore; Oakville, ON, Canada), a protease inhibitor (PI) (m250, VWR International; Radnor, PA, USA), and activated sodium orthovanadate (Calbiochem; San Diego, CA, USA). Samples were centrifuged at 4 °C for 2 min at 2000 RCF, and the resultant supernatant was centrifuged again at 4 °C for 30 min (1200 RCF).

### 2.5. Immunohistochemistry

Hearts were fixed in diastole using 1× PBS and 50 mmol KCl perfusion followed by immersion in 10% neutral-buffered formalin (VWR International) overnight. After being placed in a tissue cassette, they were dehydrated in 70, 80, and 100% ethanol sequentially and cleared in Xylene, and then paraffin wax was allowed to infiltrate them using an automated tissue processor. Samples were blocked in paraffin wax until hardened and positioned to allow 4 μm cross-sectional cuts to be made usingμ a microtome.

Paraffin sections were deparaffinized and rehydrated using xylene along with ethanol. The sections were rinsed in tap water for 5 min and then incubated in Tris-EDTA (pH 9) for 15 min using a steamer. They were then incubated in BLOXALL (Vector Labs; Newark, NJ, USA) Blocking Solution for 10 min. Sections were washed in 1× PBS for 5 min and incubated for 20 min with prediluted normal blocking serum (Vector Labs; Newark, NJ, USA). The sections were incubated for 30 min with 60 μL of Yap/Taz primary antibody (8418, Cell Signaling Technologies; Danvers, MA, USA) diluted (1:200) in a PBS-T/ 20 bovine serum albumin (BSA) (1× PBS, 0.1% Tween 20, and 1% BSA solution). Sections were then washed in PBS for 5 min. Prediluted biotinylated secondary antibodies (Horse Anti-Mouse/Rabbit IgG; Vector Labs; Newark, NJ, USA) were incubated for 30 min. The sections were washed in PBS for 5 min and incubated for 30 min in R.T.U. VECTASTAIN Elite ABC Reagent (Vector Labs; Newark, NJ, USA). The sections were then washed for 5 min in PBS and stained with DAB Peroxidase Substrate Solution (Vector Labs; Newark, NJ, USA) for 3 min under a microscope. The sections were quickly rinsed in tap water, counterstained in Hematoxylin (Vector Labs; Newark, NJ, USA) for 1 min, and washed in tap water for 5 min. Sections were dehydrated in reverse sequential ethanol and xylene solutions and then mounted using DPX Mountant for Histology (Sigma-Aldrich; Burlington, VT, USA) and allowed to dry overnight.

### 2.6. Cell Culture

H9c2 embryonic rat cardiomyoblasts from American Type Culture Collection were expanded in Dulbeco’s Modified Eagle Medium (DMEM; Gibco^®^; Waltham, MA, USA) supplemented with 10% Fetal Bovine Serum (12483020; FBS, Gibco). The passage number did not surpass 18. For experiments, cells were seeded at 500,000 cells/60 mm plate.

### 2.7. Cell Stress Treatments

*Differentiation*: H9c2 cells were differentiated into quiescent myotubes with the removal of FBS for 6 days, with media changes every 72 h (DMEM, no FBS). Cells were treated for 1 h acutely under the following conditions.

Nutrient Deprivation Solution: A 10 nM Hepes solution (Ambresco; Framingham, MA, USA) was made in Earle’s Balanced Salt Solution (EBSS, Gibco), and cells were treated for one hour.

Cytokine Stress: Tumor necrosis factor-alpha (TNFα) was supplemented (20 mg/mL) with DMEM.

Mitophagic Stress: Carbonyl cyanide-*p*-trifluoromethoxyphenylhydrazone (FCCP) [2 μM] was prepared in DMEM from a 100 mM DMSO stock solution.

Hypoxia: Cells were incubated in DMEM at 1% O_2_ using a HERAcell 150.i CO_2_ incubator (Thermo Scientific; Waltham, MA, USA).

Oxidative Stress: Hydrogen peroxide was diluted from a 0.9 M stabilized stock solution, diluted in PBS to 0.1 M, and then prepared in DMEM, and cells were treated for 24 h at [450 μM].

Ischemia: Cells were incubated at 1% O_2_ in DMEM without glucose for 24 h.

Amino Acids: L-isoleucine (J63045.14, Thermo Scientific; Waltham, MA, USA), L-valine (J62943.06, Thermo Scientific; Waltham, MA, USA), or L-threonine (J63709.30, Thermo Scientific; Waltham, MA, USA) powder were added directly to our nutrient deprivation solution to a final concentration of 105.0 mg/L and vortexed.

### 2.8. Immunoblotting

#### 2.8.1. Cell Collection and Whole-Cell Lysate Preparation

Cells were lysed in an NP-40-based lysis buffer containing 1:100 of PI, PHI, and sodium orthovanadate. The lysates were sonicated for 10 s at 20 kHz and 30% amplitude. Protein concentration was quantified using a bicinchoninic acid assay kit.

#### 2.8.2. Nuclear and Cytoplasmic Protein Extraction

After treatment media were aspirated from the cells and rinsed with PBS, a cell scraper was used to collect the resulting material into 60 μL of hypotonic buffer (10 mM Hepes, 10 mM KCl, 0.1 mM EDTA, 0.1 mM EGTA, 1 mM DTT at pH 7.9) supplemented with a 1:100 ratio of PHI, PI, and sodium orthovanadate. Lysates were placed on ice for 20 min and then subjected to centrifugation at 20,000× *g* for 5 min. The supernatant was collected as the cytosolic fraction. The pellet was resuspended in 120 μL hypotonic buffer and centrifuged again; supernatant was collected as a wash. The pellet was resuspended in 30 μL hypertonic buffer (20 mM Hepes, 0.4 M NaCl, 1 mM EDTA, 1 mM EGTA, 1 mM DTT) and left on ice for 30 min, and this process was followed by centrifugation for 5 min at 10,000× *g*. Supernatant was collected as nuclear fraction. Protein concentration was quantified using a Bradford Assay kit (Thermo Scientific; Waltham, MA, USA).

### 2.9. Western Blotting

Lysates were boiled in Laemli buffer with DTT. Samples were resolved in a 10% Mini-Protean Gel at 90 V in 1× Tris/Glycine/SDS Electrophoresis Buffer (1610772; BioRad; Hercules, CA, USA) until the dye front reached the bottom of the gel. Then, samples were transferred onto a nitrocellulose membrane (0.2μM, 1620112; BioRad; Hercules, CA, USA) at 100 V for 75 min at 4 °C in a 1× Tris/Glycine Transfer Buffer (1610771EDU; BioRad; Hercules, CA, USA). After the transfer, the membrane was washed with ddH_2_O and then stained and imaged using Reversible Protein Stain (24580, Thermo Scientific) via the ChemiDoc MP Imaging System (BioRad; Hercules, CA, USA). The stain was removed using Memcode Eraser (24580, Thermo Scientific; Waltham, MA, USA) and rinsed in ddH_2_O and then 1× Tris-Buffered-Saline-Tween 20 (TBST). Membrane was blocked for 1 h in 5% skim milk/1× TBST and rinsed in TBST before being incubated overnight at 4 °C in primary antibody. p-Yap S127 (1:1000, #13008, Cell Signaling Technologies; Danvers, MA, USA), p-Yap S397 (1:1000, 13619, Cell Signaling Technologies; Danvers, MA, USA), Yap/Taz (1:1000, 8418, Cell Signaling Technologies; Danvers, MA, USA), Lamin A/C (1:1000, 2032, Cell Signaling Technologies; Danvers, MA, USA), and GAPDH (1:1000, TA802519, Origene; Rockville, USA) were used. Afterwards, the membranes were washed in TBST and incubated in horseradish-peroxidase-conjugated secondary antibody, either anti-rabbit (1:1000-1:2000, 31460, Invitrogen, Carlsbad, CA, USA) or anti-mouse (1:1000–1:2000, Biorad; Hercules, CA, USA), with 5% milk for 2 h at room temperature. Clarity Western ECL Substrate (1705060S; BioRad; Hercules, CA, USA) luminol/enhancer and peroxide solutions were used to image membranes. Between targets, membranes were stripped in 25 mL of 0.5 M Tris-HCl/SDS buffer with 125 μL of β-mercaptoethanol for 45 min, and this process was followed by blocking. Densitometric calculations were performed using ImageLab Software v5.0 (BioRad; Hercules, CA, USA), and all targets were normalized to total protein density from the respective Memcode lane.

#### 2.9.1. Isolation and Measurement of Free Amino Acids

1 million cells were suspended in 60 μL of MilliQ water (Sigma-Aldrich; Burlington, VT, USA) and 60 μL of 2 M perchloric acid (CA71007-908, VWR; Radnor, PA, USA) along with 120 μL of internal standard (4 μg/mL) containing arginine-d7, glycine-d5, lysine-d4, and leucine-d3 (made from CDN Isotopes, D-7786, D-0277, D-2554, and D-1973, respectively) and then vortexed and lysed using an ultra sonicator for 10 s. This was followed by two minutes of sonication at room temperature, followed by 5 min in an ice bath twice to precipitate protein, and then centrifugation at 4 °C for 15 min at 13,000 rpm. The supernatant was transferred to a clean tube, and the cell pellet was washed with 60 μL of MilliQ water. A total of 150 μL of supernatant was neutralized with 120–150 μL of potassium hydroxide [2 M] (CABH9262, VWR) and then centrifuged at 13,000 rpm. Supernatant was transferred to a new tube, and the extract was freeze-dried for two to four hours, before being resuspended in 60 μL of 50:50 Methanol/ MilliQ water. Resuspended extract (10 μL) was transferred to an autosampler vial and mixed with 70 μL of Borate Buffer (186003836, Waters) for 5 min as per the Waters AccQTag Derivatization Kit (Waters Corporation; Mississauga, ON, Canada). Samples were vortexed; then, 20 μL of AccQTag Derivatization Agent (186003836, Waters Corporation; Mississauga, ON, Canada) was added, and then the samples were vortexed again and left to sit for one minute before being placed in a heating block for 10 min at 55 °C and then vortexed. Samples were run using the Waters Acquity ultra-performance liquid chromatography (UPLC) Xevo-TQS-micro–Tandem Mass Spectrometer using multiple reaction monitoring for each BCAA with respective internal standards. Results were quantified using TargetLynx (Waters Corporation; Mississauga, ON, Canada) software (version 4.2), and samples were corrected in terms of milligrams of protein.

#### 2.9.2. Quantitative Polymerase Chain Reaction (qPCR)

Cell harvesting and RNA isolation were performed using the Qiagen RNeasy Mini kit (74106, Qiagen; Hilden, Germany), and then the cells and RNA were stored at −80 °C until use. A 20 μL cDNA reaction mixture was prepared by adding a sample-specific volume equivalent to 4 μg of RNA and topping it up to a 20 μL volume with nuclease-free H_2_O that was pipetted into RNase/DNase-free strip tubes along with 20 μL of 2× Master Mix (43–688-14, Fisher Scientific; Hampton, NH, USA). The strip tubes were briefly centrifuged and then placed in a Mastercycler Nexus Gradient Thermocycler (Eppendorf; Enfield, CT, USA) for 10 min at 25 °C, 37 °C for 120 min, and 85 °C for 5 min, with a 4 °C hold. Samples were stored at −80 °C until use.

Primers were designed using OLIGO Primer Analysis Software V6.31 (Molecular Biology Insights, Inc.; Cascade, CO, USA; Table 1). Each primer set was made into a qPCR master mix, containing 5 μL of Platinum SYBR Green qPCR Supermix-UDG (11733038, Invitrogen), 0.2 μL of forward primer (10 mM), 0.2 μL of reverse primer (10 mM), 0.02 μL of ROX reference dye, and 2.58 μL of nuclease-free H_2_O. Afterwards, 8 μL of the Master Mix was added to each well in a 96-well qPCR plate, along with 2 μL of cDNA. A no-template control, containing only the master mix, was run at the same time. The plate was sealed and then centrifuged for 3 min at 2500 rpm. The reaction was incubated at 50 °C for 120 s and 95 °C for 120 s, and there were 35–40 cycles of incubation at 95 °C for 3 s and 60 °C for 30 s (Applied Biosystems Viia 7; Thermo Scientific; Waltham, MA, USA). All qPCR data were analyzed as per Minimum Information for Publication of Quantitative Real-time PCR Experiments (MIQE) guidelines. We used two reference genes that were not influenced by culture conditions or treatments for group effect comparison (Supplemental Figure S3). Data were analyzed using Igfbp3 as a reference gene and confirmed by a second reference gene, Hprt1, to compare nutrient deprivation to that of the DMEM controls, and Igfbp3 and B2m were used as reference genes to allow comparison between nutrient deprivation for + L-isoleucine and the DMEM controls.

### 2.10. Immunocytochemistry

Cells were seeded (600,000/60 mm plate) on a glass coverslip and differentiated (protocol above), and then respective treatments (control vs. nutrient deprivation) were conducted. Cells were washed with 1× PBS and then fixed using warmed formaldehyde (4% in PBS) for 5 min. The cells were washed three times with ice-cold PBS before being permeabilized in 0.1% Triton-X-100 for 15 min and then washed again three times in PBS. Cells were then blocked using a 1% BSA in 1× PBS 0.1% Tween 20 solution for 15 min, and then another wash was performed. Cells were then incubated in AlexaFluor 488 Phalloidin (Thermo Scientific, diluted 1:1000) stain in 1× PBS for 30 min while protected from light; then, they were washed and incubated in primary antibodies diluted 1:400 at 4 °C for 24 h. After being washed, they were incubated in a fluorophore-conjugated secondary antibody (Alexa Fluor 647 donkey-anti-rabbit, diluted 1:400) for 30 min and then washed again. Cells were then stained with Hoechst 33342, which was diluted 1:3000 in 1× PBS for 1 min, and washed one last time before the coverslip was mounted on a glass slide using 1:1 glycerol and PBS, sealed with clear nail polish (at the edges), and allowed to dry overnight at 4 °C.

### 2.11. Statistical Analysis:

All results are representative of the means ± standard deviations. Statistical analysis was performed using GraphPad Prism 9 (GraphPad Software Inc; San Diego, CA, USA). Pairwise comparisons were performed using a Student’s two-tailed *t*-Test, while any results that had three or more groups were analyzed using a one-way or analysis of variance (ANOVA). Statistical significances were measured using Tukey’s post hoc test. *p*-values < 0.05 were considered statistically significant.

## 3. Results

### 3.1. Yap Signalling in Myocardial Infarction and Associated Stressors

**Figure 1:**
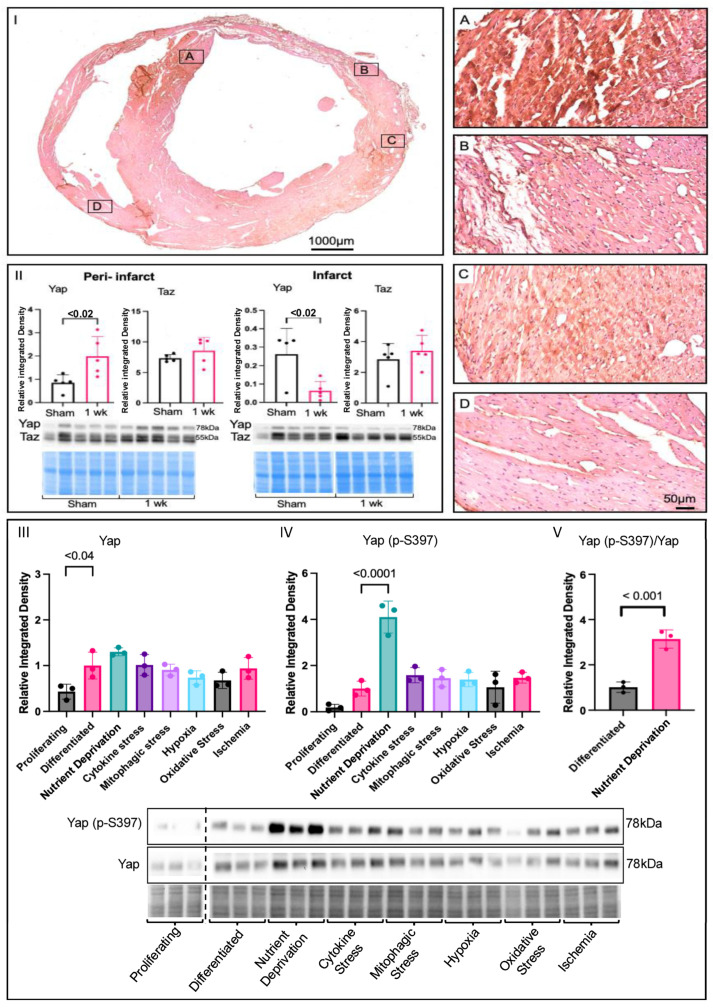
Changes in yap signalling under ischemic conditions. (**I**) Yap immunohistochemistry of a 28-day-post-MI mouse heart. Peri-infarct zone (**A**–**C**) had higher Yap expression, shown by the brown, positive Yap signal, compared to the remote region, (**D**) which was mostly pink. (**II**) Yap expression was increased in the peri-infarct zone and decreased in the infarct zone of 1-week-post-MI mice compared to shams. Immunoblot and densitometric analysis of Yap and Taz peri-infarct and infarct zones of shams compared to 1-week-post-MI mice. (**III**) Analysis of Yap in screening of proliferating cells (DMEM + 10% FBS), differentiated cells (DMEM), and cells that experienced metabolic shock (EBSS, 1 h), cytokine stress (TNFα 20 ng/mL, 12 h), mitophagic stress (FCCP 2 μM, 12 h), hypoxia (O_2_ 1%, 24 h), oxidative stress (H_2_O_2_ 450 μM, 24 h), and ischemia (O_2_ 1%, + 0% glucose, 24 h) in H9c2 cells, where expression was higher in differentiated cells compared to proliferating ones. (**IV**) Yap (p-S397) levels were significantly higher under metabolic shock conditions than under those induced by all other ischemic stressors. (**V**) Significant increase in the ratio of Yap (p-S397) to total Yap under nutrient deprivation conditions, showing corresponding relative blots and Pierce Memcode to demonstrate uniform loading.

To determine the effects of MI on Yap signalling in cardiac remodeling, immunohistochemistry was performed on 28-day-post-MI mouse hearts, and the results were compared to those obtained for the shams. Positive Yap-staining was observed and localized mainly in the peri-infarct zone post-MI (Figure 1I-A,B), as opposed to the remote region, and, to some extent, in the remote ventricles (Figure 1I-C,D). An increase in Yap levels was detectable via Western blot as soon as 7 days post-MI in the peri-infarct zone, with no change in the infarct zone, suggesting it regulates early and late remodelling. Taz—a paralog of Yap—levels remained unchanged in the peri-infarct and infarct zones compared to the shams (Figure 1II). To determine how Yap changes in response to specific MI stress patterns associated with ischemia, we used cardiomyocyte-like (H9c2) cells as a model and screened several commonly associated drivers (nutrient deprivation and cytokine-related, mitophagic, hypoxic, oxidative, and ischemic stress). We found that total Yap expression was higher in differentiated cells compared to proliferative cells; however, the total Yap levels were unchanged under ischemic stressor/stimulus conditions in differentiated cells (Figure 1III). As phosphorylation is important for Yap signalling, we examined one of the key phosphorylation sites (p-S397) under the various conditions we induced. Only acute nutrient deprivation (modelling total occlusion) resulted in a significant increase in Yap (p-S397) (Figure 1IV). The increase in Yap (p-S397) was confirmed by the ratio of Yap (p-S397) to total Yap (Figure 1V). Similar results were also found, with a second key phosphorylation site on Yap (p-S127) (Supplemental Figure S1). These findings suggest that Yap signalling is most sensitive to severe metabolic stress associated with MI and that nutrient deprivation has a large impact on the phosphorylation of Yap.

### 3.2. Nutrient Deprivation Affects Yap Post-Translation Modification and Compartmentation

**Figure 2:**
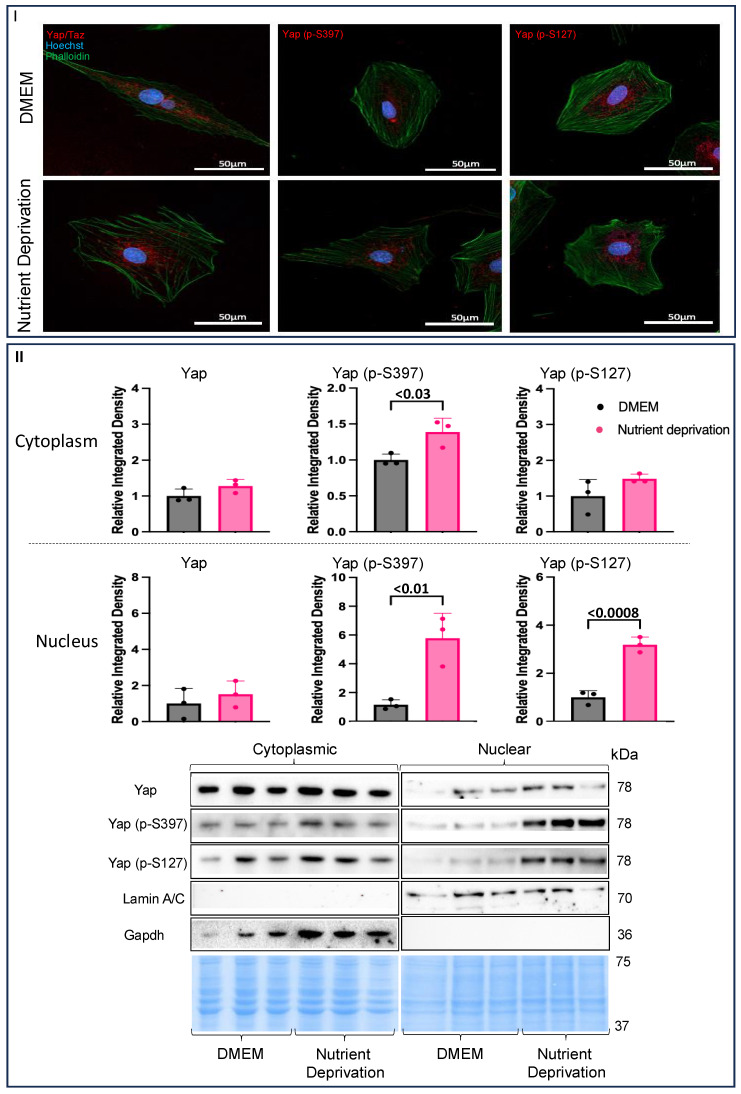
Evidence of nuclear compartmentalization under nutrient deprivation conditions. H9c2 cells were differentiated for 6 days in the absence of FBS. On the day of the experiment, the media for the controls were changed, and our treatment groups were administered nutrient deprivation solution for 1 h. Cells were permeabilized and blocked and then stained with Phalloidin and Hoechst and incubated with respective fluorescent antibodies for immunofluorescence. (**I**) There were no changes in Yap compartmentalization (nuclear and cytoplasmic) compared to what was observed for the controls. Yap (p-S397) and Yap (p-S127) levels were higher in the nucleus under metabolic shock conditions compared to the levels observed for the controls. (**II**) Cytoplasmic Yap expression was unchanged under nutrient deprivation conditions compared to that for the controls. (**II**) Yap (p-S397) expression was increased in the cytoplasm, while Yap (p-S127) was unchanged under nutrient deprivation conditions compared to the controls. Nuclear Yap expression was unchanged compared to controls. Nuclear Yap (p-S397) and Yap (p-S127) expression were both increased in nutrient deprivation conditions compared to controls. Representative blots for Yap, Yap (p-S397), and Yap (p-S127) and nuclear and cytoplasmic markers, Lamin A/C and Gapdh, respectively, and Pierce Memcode demonstrating uniform protein loading.

Immunohistochemistry was used to investigate whether nutrient deprivation affects the translocation of Yap. The total Yap levels did not change, and there was little indication of migration or translocation to the nucleus compared to what was observed for the controls. Yet, both Yap (p-S397) and Yap (p-S127) appeared to cluster towards the nucleus (indicated by speckling and a purplish colour of the nuclei) to a greater degree under nutrient deprivation conditions compared to the controls (Figure 2I). Nuclear and cytoplasmic fractionation was performed to substantiate the translocation of Yap and phosphorylated Yap to the nucleus. There were no changes in total Yap levels in either fraction compared to the controls, a finding that is in agreement with immunohistochemistry results. However, Yap (p-S397) was significantly increased in the nucleus and the cytoplasm, while Yap (p-S127) was increased only in the nucleus compared to the controls (Figure 2II), suggesting that acute nutrient deprivation plays a role in the translocation of phosphorylated Yap.

### 3.3. Amino Acid Nutrient Sensitivity Impacts Yap Signalling

**Figure 3:**
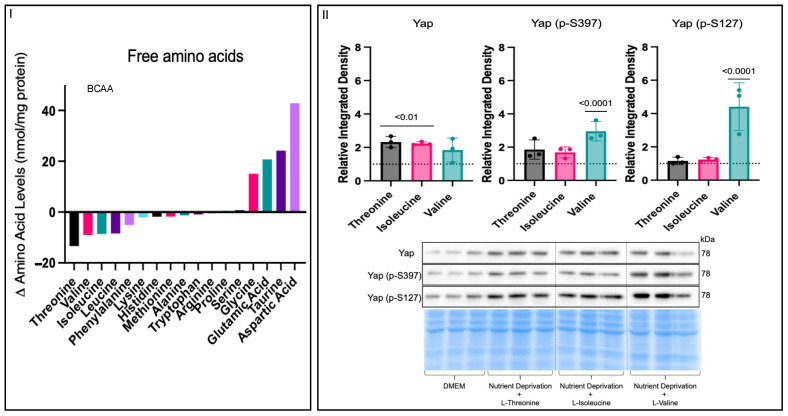
Effects on free amino acids under nutrient deprivation conditions. H9c2 cells were differentiated for 6 days in the absence of FBS. On the day of the experiment, the media for the controls were changed, and our treatment groups were administered nutrient deprivation solution for 1 h. (**I**) UPLC-determined free amino acid level differences between controls and cells under nutrient deprivation conditions. All non-essential amino acid levels increased, while all essential amino acid levels decreased, under nutrient deprivation compared to the controls. All three branched-chain amino acid levels were decreased to the same extent. (**II**) On the day of the experiment, the media for the controls were changed, and our treatment groups were administered nutrient deprivation solution with respective amino acids supplemented for 1 h before harvest. Total Yap expression was increased in threonine and isoleucine groups compared to that for the DMEM controls (dashed line), while there was no change between the controls and cells given valine supplementation. Supplementation of threonine and isoleucine caused no changes in Yap (p-S397) expression compared to DMEM controls. Yap (p-S397) expression was increased in the valine group compared to that for the controls. Yap (p-S127) expression was unchanged between the threonine and isoleucine groups and controls and increased in the valine treatment compared to that for the controls. Representative blots for Yap, Yap (p-S397), and Yap (p-S127) and Pierce Memcode demonstrating uniform protein loading.

Nutrient deprivation is one component of ischemia, along with other components, such as hypoxia and oxidative stress (Supplemental Figure S2I). Hypoxia and oxidative stress conditions had no effects on Yap signalling, unlike nutrient deprivation (Figure 1III,IV), which increased phosphorylated Yap levels compared to those for the controls. Nutrient deprivation is defined as an acute state where cells are deprived of the three essential macronutrients: glucose, fatty acids, and amino acids. To establish which of the three essential macronutrients was responsible for the increase in phosphorylated Yap levels, we used reductive reasoning (Supplemental Figure S2I–VI to determine that amino acids were likely driving the changes in Yap signalling under nutrient deprivation conditions. To better understand the effects of nutrient deprivation conditions on amino acids, we characterized the changes in free amino acids under nutrient deprivation conditions compared to the controls via UPLC. Free essential amino acid levels were decreased, while non-essential free amino acid levels were higher under nutrient deprivation conditions compared to those for the controls (Figure 3I). Furthermore, nutrient deprivation conditions decreased all branched-chain amino acid levels to the same extent compared to the controls. To determine which amino acids could attenuate the increase in phosphorylated Yap under nutrient deprivation conditions, we chose the three amino acids whose levels were most significantly decreased and supplemented them into the nutrient deprivation conditions. Concentrations equivalent to the DMEM control media (0.8 mM) of the three amino acids L-threonine, L-valine, and L-isoleucine were added separately under the nutrient deprivation conditions. Only supplementation with L-threonine and L-isoleucine increased total Yap levels, whereas only supplementation with L-valine increased phosphorylated Yap S397 and S127 levels (Figure 3II). The attenuation of phosphorylated Yap S397 and S127 levels following the supplementation of L-threonine and L-isoleucine suggests that the absence of these amino acids could be responsible for the increase in phosphorylated Yap levels following nutrient deprivation (Figure 1IV). To further narrow down our focus, we considered that threonine is catabolized into isoleucine and regulated by an inhibitory feedback mechanism, where isoleucine binds allosterically to threonine deaminase (the first enzyme in the catabolism pathway) when isoleucine levels are high, thereby inhibiting the binding of threonine. In contrast, when isoleucine levels are low, threonine can bind, and the catabolism to isoleucine can continue, suggesting that isoleucine is a limiting factor [21]. Since the relationship between threonine and isoleucine relies on isoleucine as a limiting factor, all further experiments were performed with L-isoleucine.

**Figure 4:**
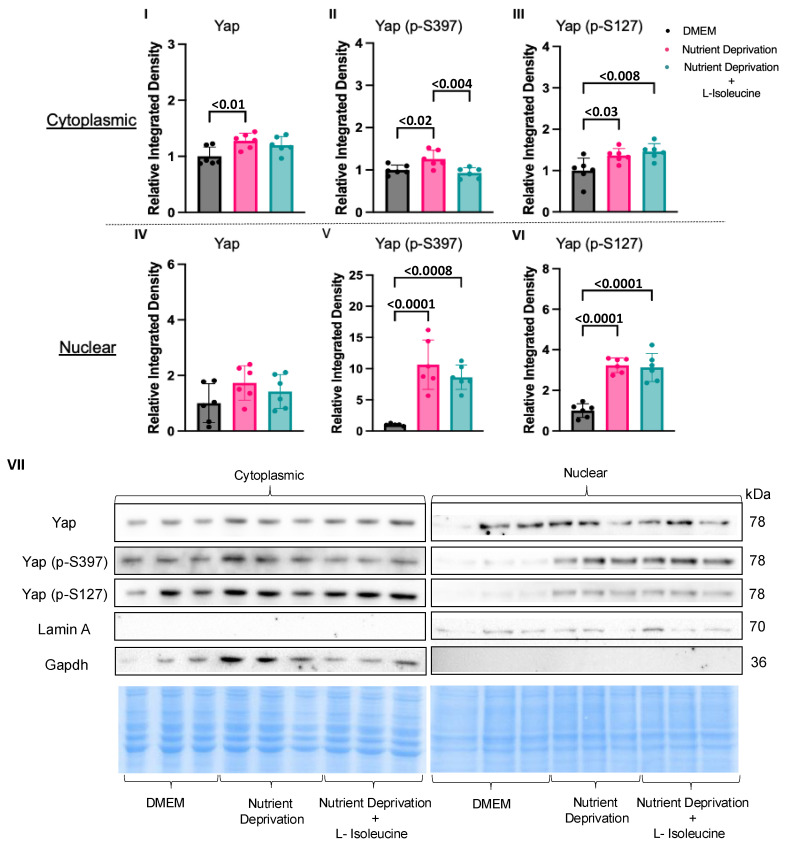
L-Isoleucine decreased cytoplasmic Yap (p-S397) levels back to DMEM control levels. H9c2 cells were differentiated for 6 days in the absence of FBS. On the day of the experiment, the media for the controls were changed, and our treatment groups were administered nutrient deprivation solution or nutrient deprivation solution with L-isoleucine for 1 h and subsequently subjected to nuclear and cytoplasmic fractionation. (**I**) Nutrient deprivation increased Yap expression compared to that observed for the controls; however, expression was unaffected between the controls and the cells subjected to nutrient deprivation with L-isoleucine. (**II**) L-isoleucine supplementation decreased cytoplasmic Yap (p-S397) levels compared to nutrient deprivation alone, while it did not affect cytoplasmic Yap levels (p-S127) (**III**) or any of the nuclear fractions for Yap, Yap (p-S397), and Yap (p-S127) (**IV**–**VI**). Representative blots for Yap, Yap (p-S397), and Yap (p-S127) and nuclear and cytoplasmic markers, Lamin A/C and Gapdh, respectively, and Pierce Memcode demonstrating uniform protein loading (**VII**).

Sub-cellular fractionation was used to determine the effects of L-isoleucine supplementation on the translocation patterns of Yap and phosphorylated Yap to/from the nucleus. Interestingly, with additional data points, the total Yap levels were increased under nutrient deprivation conditions compared to those for the DMEM controls in the cytoplasmic fraction, though the effect size was small (Figure 4I). Total Yap levels were unchanged between the DMEM controls and the cells subjected to nutrient deprivation + L-isoleucine in the cytoplasm (Figure 4IV). Supplementation with L-isoleucine attenuated the increase in cytoplasmic phosphorylated Yap S397 levels seen under nutrient deprivation conditions alone compared to that observed for the DMEM controls (Figure 4II). However, supplementation with L-isoleucine did not affect phosphorylated Yap S397 and S127 levels in the nuclear fraction or Yap S127 in the cytoplasm compared to nutrient deprivation alone (Figure 4III,V,VI). These findings suggest that L-isoleucine has a major role in the phosphorylation patterns of Yap S397 but does not reverse all effects of nutrient deprivation and its elicited compartmentation.

### 3.4. Yap-Associated Gene Targets Expression Relevant to Myocardial Infarction

**Figure 5:**
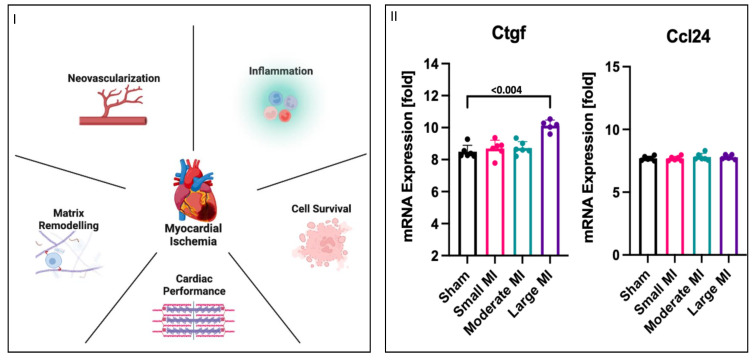

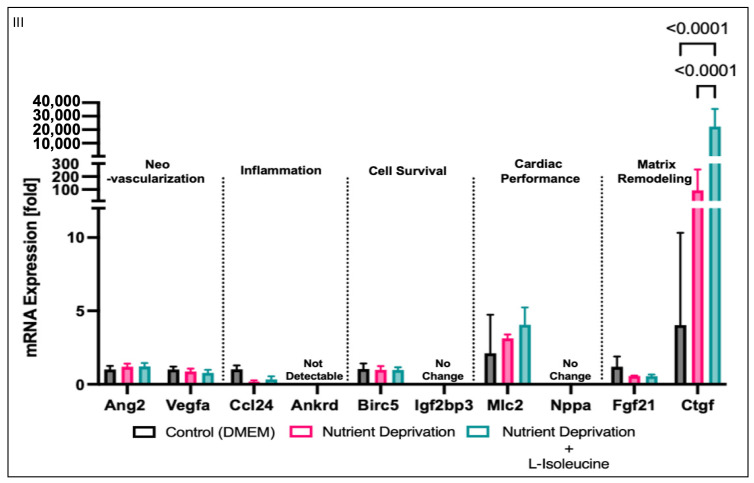
Increase in mRNA expression of matrix-related genes. (**I**) Myocardial infarction has five categories associated with recovery after an MI: neo-vascularization, inflammation, cell survival, cardiac performance, and matrix remodelling. (**II**) mRNA (log2-transformed) was analyzed in rat MI model using public microarray datasets, wherein left ventricles were removed from rats that were subjected to left coronary artery ligation to induce large, moderate, or small myocardial infarctions. Genes associated with Yap were identified as *Ctgf* and *Ccl*24. *Ctgf* levels were significantly higher in the large-MI group compared to those for all other groups, while *Ccl*24 levels did not vary between all four groups, including the control shams. (**III**) H9c2 cells were differentiated to determine any similar associations with AMI after nutrient deprivation with or without L-isoleucine for 1 h. Two genes that had a priori links to Yap regulation from each MI recovery category were tested, and no changes in genes associated with neo-vascularization, inflammation, cell survival, or cardiac performance were observed. However, the levels of *Ctgf*, which is associated with matrix remodelling, were significantly increased under nutrient deprivation with L-isoleucine compared to nutrient deprivation alone.

Changes in phosphorylation patterns and Yap at the protein level could lead to alterations in downstream Yap-mediated gene expression. Therefore, we wanted to investigate the effects of nutrient deprivation and nutrient deprivation + L-isoleucine on gene expression. To simplify a targeted search for qPCR-plausible Yap-mediated gene targets, we first considered five classical categories of MI recovery: neovascularization, inflammation, cell survival, cardiac performance, and matrix remodelling. We accessed an open-access microarray dataset from GEO to screen for any Yap-associated genes in MI but found that only two were listed in the dataset (dataset: GDS4907), connective tissue growth factor (*Ctgf*) and c-c motif chemokine ligand 24 (*Ccl24*). *Ctgf* expression was significantly increased in the large-MI group compared to that for all the other groups, while there was no change in *Ccl*24 expression between any of the groups (Figure 5II). For each MI category, we performed a simple qPCR screen of two Yap-associated genes that aligned with a category of MI remodelling. Our qPCR results showed no significant changes in any Yap-associated genes related to four of the MI recovery themes: neovascularization, inflammation, cell survival, or cardiac performance. However, as with the GEO dataset, the levels of *Ctgf*, a matrix-associated gene, were significantly higher under nutrient deprivation than the levels observed for the controls. Yet, the addition of L-isoleucine did not attenuate *Ctgf* expression; rather, it further increased expression. Collectively, these results suggest that Yap-associated *Ctgf* expression could be a downstream target selected in response to nutrient deprivation conditions caused by MI and proportional to the size/severity of infarction.

## 4. Discussion

Several studies suggest Yap signalling can improve outcomes after an MI [16,22,23]; however, the molecular drivers and mechanisms that regulate Yap and its effects in cardiac-related cells post-MI are less understood. The purpose of this study was to determine the stressors and molecular mechanisms that may regulate and/or promote Yap signalling after an MI. We observed increased Yap levels in the infarct and peri-infarct zone after MIs, which could be explained by an increase in Yap levels in cardiomyocytes or fibroblasts after an MI, as Yap expression has been seen to increase in cardiac fibroblasts three to five days after an MI [24]. Additionally, we found that amongst simplified ischemic stress patterns associated with MI in H9c2 myotubules, Yap signalling was particularly sensitive to the deprivation of essential nutrients (glucose, fatty acids, and amino acids). Our data then resolved that this was predominately influenced by amino acids. Lastly, the changes in Yap signalling caused by acute nutrient deprivation affected the Yap-associated gene Ctgf. Collectively, these findings improve our understanding of some of the underlying mechanisms that are involved in Yap signalling in adult cardiomyocytes and may provide insight into potential therapeutic targets that could improve remodelling after an MI.

### 4.1. The Effects of Nutrient Deprivation on Yap Signalling

Yap signalling and methods of regulation have been extensively studied in relation to diverse cell types and disease conditions, but the specific molecular mechanisms underlying the effects of Yap activation after an MI are not fully understood. While mechanical stress is considered the primary driver of cardiac Yap signalling [15,25,26], this study reveals that cardiac metabolic stress can also be a major driver of altered Yap signalling. The role of Yap signalling—in predominantly cancer metabolism—is to regulate cellular proliferation and substrate utilization [27]. When energy is low, Yap is inhibited by phosphorylation. In times of hypertrophy or proliferation, the absence of essential amino acids could also, reasonably, act as a checkpoint for Yap signalling, restricting growth. There is little clarity, however, on Yap-signaling in the context of non-proliferating cardiomyocytes, or models of the same, and what drives observed changes. Our investigation using cardiomyotubes revealed that acute nutrient deprivation, more than any other constituent of ischemia, was a salient cause of Yap phophoregulation and nuclear accumulation. These results are surprising, as many other stressors have been identified as potential effectors of Yap signalling in the heart, but this could be due to a lack of cellular specificity amongst a diverse pool of cardiac cells (cardiomyocytes, fibroblasts, vascular cells, etc.). Acute nutrient deprivation did not change total Yap levels, yet it did increase post-translational modification at Yap (p-S397) and Yap (p-S127) in whole-cell lysate—consistent with the inactivation and cytoplasmic restriction of Yap signalling—and, therefore, is not likely to result in increased gene transcription of downstream targets. In determining which essential nutrient was responsible for the acute changes in Yap, initially, glucose and/or fatty acids were postulated as being responsible since they are major energy sources for cardiomyocytes and have previously been linked to the phosphorylation of Yap, leading to its inactivation [27]. We found that glucose and fatty acid supplementation alone did not reverse the increase in Yap (p-S397) and Yap (p-S127) modifications after nutrient deprivation conditions were imposed, thus implicating amino acids as the nutrient responsible. Further analysis of cells with nutrient deprivation revealed a shift in amino acid bioavailability, with non-essential amino acid levels having increased but essential amino acid levels having decreased. This could be explained by the fact that essential amino acids, by necessity, come from extrinsic sources, whereas non-essential amino acids can be obtained via proteolysis, including in excess should the purpose be to extract and recycle essential amino acids. Therefore, in response to acute nutrient deprivation, the cardiomyocytes may quickly consume the essential amino acids to maintain appropriate function and dispense with proteins to build up excess non-essential amino acids thereafter. Of the essential amino acids, the levels of threonine were decreased to the greatest extent, followed by those of valine, isoleucine, and leucine—three branched-chain amino acids. Supplementation with threonine and L-isoleucine returned phosphorylated Yap signals to the control levels in whole-cell lysate. We then focused on isoleucine as a rate-limiting amino acid, given there exists a feedback loop between threonine and isoleucine formed from threonine deaminase (the enzyme involved in catalyzing the first reaction, which is inhibited by the allosteric binding of isoleucine to inhibit the reaction at high levels of isoleucine) [21]. As such, increased levels of threonine can be generated by its conversion into isoleucine. Additionally, the levels of these three branched-chain amino acids were decreased to the same extent, which may be explained by the fact that the same enzymes metabolize all three [28]. These findings highlight the critical role of amino acid bioavailability, particularly regarding isoleucine, in modulating Yap signalling in cardiomyocytes under nutrient deprivation conditions.

### 4.2. Compartmentalization of Yap in Rat Cardiomyotubes after Nutrient Deprivation

Canonically, the translocation of Yap between the nucleus and cytoplasm is a critical mechanism regulating its activity. To investigate the effects of acute nutrient deprivation, we sub-fractionated the nuclear and cytoplasmic compartments of H9c2 cardiomyotubes. Consistent with our whole-cell lysate results, nutrient deprivation increased the phosphorylation of Yap at S397 and S127 in both the nucleus and cytoplasm compared to that observed for the controls. However, this is atypical, as most reports suggest that phosphorylated Yap is cytoplasmically retained, degraded, or associated with nuclear export to the cytoplasm [29,30]. It has been suggested that there could be a biphasic pattern of localization for Yap signalling once Yap is phosphorylated [31] that is time-dependent [32]. This might account for the increase in total Yap expression in the cytoplasm under nutrient deprivation conditions compared to the controls, but our study was time-constrained to just 1 h, and we did not account for all post-translational modifications of Yap that could occur and affect binding partners that either promote or restrict gene expression. It may, therefore, be reasonable to hypothesize that if the time course were expanded, there would be a flux of Yap from the nucleus to the cytoplasm. Interestingly, Yap and Taz do not have a nuclear localization signal, which is typically responsible for the accumulation of proteins in the nucleus. Therefore, the exact mechanism of their nuclear shuttling remains unclear. However, there is evidence that mechanical forces, in addition to biochemical cues, can regulate Yap translocation, particularly in the absence of cell–cell contact, as seen after a cardiac injury [33]. Future studies in which Yap is immunoprecipitated under these conditions could identify key regulatory binding partner proteins necessary for compartmentation or cytoplasmic/nuclear shuttling.

Supplementation with the essential amino acid L-isoleucine maintained Yap, p-Yap (S397), and p-Yap (S127) levels in proportions comparable to those observed for the controls in the whole-cell lysates. Yet, in the subcellular fractions, isoleucine supplementation only returned cytoplasmic p-Yap (S397) to control levels. In the nuclear compartment, p-Yap (S397) levels remained higher than those for the controls. Further, p-Yap (S127) levels also remained higher in both cytoplasm and nuclear fractions even in the presence of isoleucine (Table 1). Typically, phosphorylation at S397 and S127 is associated with the inhibition of Yap-associated transcription, as it is often associated with the export of Yap to the cytoplasm, a process that subsequently leads to proteasomal degradation [11]. We sought to determine if whole media were able to restore compartmentation to a greater degree than the controls; we observed that cells that were refed normal media for an hour actually accelerated Yap-degradation and left S127 Yap levels elevated as a ratio of total Yap (Supplemental Figure S1), suggesting that time and substrate are not independent mechanisms. Our data are not fully time-resolved and did not account for the canonical or noncanonical sites of regulation. The fact that a classically Yap-associated gene, *Ctgf*, was elevated where S127 was consistently phosphorylated, even in the presence of isoleucine, suggests that S127 may be associated with increased gene expression under some circumstances, either by nuclear translocation or retention. Further studies will be required to resolve the nature of Yap shuttling.

### 4.3. Connective Tissue Growth Factor, Yap Signalling, and the Heart

The *Ctgf* gene encodes the Ctgf protein, which is also known as cellular communication network factor 2. The regulation of Ctgf can occur at the transcriptional, post-transcriptional, and translational levels and is controlled by several pathological and physiological cues. As a secreted protein, it initiates signal transduction by binding to various extracellular constituents and receptors (e.g., integrins, heparan sulfate proteoglycans, LRPs, and TrkA). The consequences of Ctgf binding are multifaceted, including effects on the extracellular matrix (ECM), the endothelial–mesenchymal transition, macrophage polarization, autophagy, and senescence-associated metabolic disruption [34]. It is responsible for the direct binding of cytokines and mediates ECM-related proteins, in addition to regulating growth factor and cytokine activity by modulating crosstalk between signalling pathways. Ctgf plays a central role in fibrosis by controlling fibroblast proliferation, angiogenesis, matrix production, and ECM deposition. Fibroblasts are fundamental for creating and maintaining the ECM, and their migration, activation, and differentiation into myofibroblasts are major drivers of fibrosis after injury. Ctgf acts as a downstream mediator of transforming growth factor-β (TGF-β), which regulates myofibroblast differentiation and tissue formation. The release of Ctgf from M2 macrophages is also associated with the proliferation and migration of fibroblasts, contributing to ECM formation [35]. In addition to its effects on fibroblasts and macrophages, Ctgf plays a role in endothelial cell function and angiogenesis. Ctgf increases the production of vascular endothelial growth factor (VEGF), which is responsible for angiogenesis, through various pathways (e.g., AKT, ERK, and Pi3K) that increase miR-210 levels. Upregulation of miR-210 leads to the inhibition of prolyl hydroxylase 2 activity, which promotes increased VEGF expression and angiogenesis [36]. The expression of *Ctgf* is controlled by various transcription factors, including the TEAD transcription factor and its coactivator Yap [34]. Of the Yap-associated genes we tested, only the expression of *Ctgf* was markedly changed in response to nutrient deprivation conditions and increased again when L-isoleucine was supplemented. While there was no change in total Yap expression following isoleucine supplementation, there was a decrease in the inhibitory phosphorylation of Yap at S397 in the cytoplasm compared to that observed with nutrient deprivation alone. This suggests that a reduction in Yap phosphorylation may result in the activation of Yap, allowing for the transcription of *Ctgf*. The activities of Yap/Tead and Ctgf are mutually regulated, suggesting that increased activated Yap levels (unphosphorylated) are correlated with increased *Ctgf* [37]. The role of Yap in *Ctgf*-associated angiogenesis was confirmed by the re-expression of Yap, which rescues angiogenesis in *Ctgf* mutant mice [37]. This suggests that Ctgf itself may be a feedback regulator of Yap signalling.

As for fibroblasts, activated Yap increases Ctgf expression to promote fibroblast proliferation [38]. Yap also plays a role in regulating the ECM through Ctgf expression; for example, in the heart, after an MI, elevated unphosphorylated Yap levels coincide with increased ECM production. This was confirmed using verteporfin, a drug that inhibits the binding of Yap to TEAD to arrest gene transcription and decreases ECM production and proliferation of fibroblasts. The supplementation of this drug, therefore, resulted in decreased fibrosis and stiffness of the heart after an MI compared to the controls [39]. Inhibition of Ctgf might improve survival and ejection fractions in addition to improving remodelling by increasing the cross-sectional area of cardiomyocytes, heart weight, and fibrotic gene expression [40]. Upregulation of Ctgf has also been associated with the hypertrophy of cardiomyocytes [41]. The role of Yap-associated Ctgf is likely cell- and time-dependent and may not be readily reversed, as we showed through simple L-isoleucine supplementation. Its effects on all cardiac cell types are diverse and include both autocrine and paracrine effects likely to influence all major remodelling events after MI, but its activation is one of the most rapid responses detected during cardiac stress.

### 4.4. Clinical Relevance

In a clinical setting, elevated circulating BCAA levels after an MI are associated with an increased risk of cardiovascular mortality and acute heart failure, particularly for patients with an ST-elevation MI requiring reperfusion [42]. However, this BCAA elevation is not universal, as some studies found decreased circulating BCAA levels post-MI despite increased cardiac BCAA levels due to impaired BCAA catabolism in mice. Wang et al. suggest that a permanent MI is correlated with increased cardiac BCAAs caused by defective catabolism [43]. This impaired BCAA catabolism has been linked to a progressive loss of cardiac contractile capacity and early death in animal models [44]. Gannon et al. suggest that in conditions where energy is deprived, BCAA might improve sensitivity to glucose uptake. In conditions with excess energy, the catabolism of BCAA is disrupted, which leads to the accumulation of BCAA either in circulation or intracellularly [45]. The regulation of BCAA metabolism appears complex, and there is evidence that Yap plays an important role, wherein decreased Yap activity is correlated with lower levels of the amino acid transporter SLC7A5, leading to higher intracellular amino acid levels [46]. This is consistent with our results that show the supplementation of L-isoleucine amidst our nutrient deprivation conditions resulted in decreased Yap (p-S397) levels in the cytoplasm compared to nutrient deprivation alone and increased *Ctgf* expression, the latter of which has been associated with poor remodelling and increased death after an MI. Interestingly, Yap activation (unphosphorylated) upregulates SLC7A5 and other nutrient uptake genes, in addition to provoking an upregulation of *Ctgf* [47]. These findings suggest there may be a potential mechanism between Yap signalling and amino acids where in Yap acts to regulate amino acid metabolism instead of amino acids acting upon Yap signalling, or perhaps it operates in both directions. Given the link between elevated BCAA levels and poor outcomes and the fact that activating Yap signalling can decrease amino acid levels by increasing SLC7A5 activity, increasing Yap signalling after MI may promote better outcomes for patients by improving adaptive remodelling. Therefore, these data highlight a relationship between Yap signalling and amino acids, yet the full mechanism needs to be explored further.

## 5. Limitations and Future Directions

While this study provides an observational overview of the relationship between Yap signalling and amino acids, there are limitations to this work that limit the opportunity to make definitive conclusions or understand the corresponding mechanisms fully. Firstly, this study was predominantly performed *in vitro* using H9c2 cardiomyocytes. Although *in vitro* studies allow for cell specificity and the direct modelling of conditions for the identification of molecular mechanisms, they lack the ability to reveal the complexity of the humoral, cellular, and molecular mechanisms interacting amongst various cardiac cell types that are fully matured and functioning to meet physiological demands. Furthermore, our results stem from one cell line; comparing them to those obtained using another cell line, such as AC16s (human cardiomyocytes) or isolated primary cardiomyocytes, could support our conclusions, provide a more complete picture of the mechanisms involved, and account for different species effects. Additionally, our short timeline of one hour could be a limitation of our study as it provides only a snapshot in time; it does not resolve what occurs with respect to integrative signalling and temporal progression with an MI. Though we were able to mimic many drivers of MI in our study, we lacked a model of waste accumulation that occurs after MI. Therefore, its role in Yap signalling was not identified in this work. Another limitation of our study is that we only measured free amino acid concentrations. However, both circulating and intracellular BCAA play an important role in remodelling after an MI. Additionally, our experiments were only carried out by testing the supplementation of only three amino acids, and they were all tested separately, yet combinations of amino acids might influence cell signalling in variable ways that we have not determined. Leucine could be of particular interest since isoleucine and leucine have the same chemical formula but slightly different chemical structures. Leucine in particular is implicated in the regulation of other pathways, such as mTOR, and there has been an association between mTOR activity and vascular growth that is YAP/TAZ-dependent [47]. Despite these limitations, we have put forward evidence for the amino-acid-regulated signalling of Yap that can potentially influence remodelling in the context of MI. This is a potentially significant hypothesis-generating study that can lead to further cell-regulated and time-specific *in vivo* analysis that analyzes metabolic changes as both causes and effects in MI outcomes. Moreover, the compartmentation of Yap and its post-translational modifications should be further explored in terms of promoting or restricting gene expression patterns, particularly with unbiased analysis tools.

## 6. Conclusions

Our study provides insights into Yap signalling, showing for the first time that acute nutrient deprivation conditions increase Yap phosphorylation and compartmentation between the nucleus and the cytoplasm. We show the importance of amino acids as regulators of Yap signalling in a cellular model of cardiomyotubes. Furthermore, our findings indicate there is a relationship between L-isoleucine, Yap, and *Ctgf* gene expression in early remodeling where acute nutrient deprivation and amino acid imbalance may occur. Collectively, these results should promote further exploration and understanding of the crosstalk between amino acid signaling, including that for isoleucine, and Yap-mediated heart remodelling. Integration with other signalling cascades could lead to the recognition of potential therapeutic targets that might improve cardiac remodelling, a novel lead for improving patient outcomes.

## Figures and Tables

**Table 1:** Primer sequences and annealing temperatures of oligonucleotides used in qPCR.

Target		Primer Sequence (5′-3′)
RtAng2	Forward	TCAGCACTATGATGCCAAGCC
	Reverse	TTGATGCTGCCCTTATTGCCAT
RtAnkrd1	Forward	GGCCAGCTCCAGGGGTTCAGC
	Reverse	GCTGAACCCCTGGAGCTGGCC
RtBirc5	Forward	TTCCTTACAGTCAAGAAGCAGGT
	Reverse	TTCTTGGCTCTTTGTTTGTCCA
RtB2m	Forward	ACATCCTGGCTCACATGAA
	Reverse	ATGTCTTCGGTCCCAGGTG
RtCcl24	Forward	CTCTAAGAAGCAGTTCAAGGCTA
	Reverse	ACCTCAAATTTTCTATGTGGCTA
RtCtgf	Forward	CGCTGACATTCTGATTCCAGT
	Reverse	CTGATCCATTGCTTTACCGTCT
RtGapdh	Forward	GGCCGAAGGGCCCACTA
	Reverse	TGTTGAAGTCACAGGAGACAACCT
RtHprt1	Forward	CCCAGCGTCGTGATTAGTGATG
	Reverse	TTCAGTCCTGTCCATAATCAGTC
RtIgf2bp3	Forward	ATCCCCTTGAAGATTTTAGCTC
	Reverse	ATTTTAGTGTCCGTGTCTTGC
RtMlc2	Forward	TCAAAGTCTGTTCCGTCCCT
	Reverse	AACTTGGCGTCCATAATTGCT
RtNppa	Forward	CGGACAAAGGCTGAGAGAGAA
	Reverse	TTCTCTCTCAGCCTTTGTCCG
RtU6	Forward	GCTTCGGCAGCACATATACTAA
	Reverse	AACGCTTCACGAATTTGCGT
RtVegfα	Forward	TGGTGCTACTGTTTATCCGTA
	Reverse	ATTATCTCGGAAAACTGCTCT
Rtβ-actin	Forward	CGAGTACAACCTTCTTGCAGC
	Reverse	ACCCATACCCACCATCACAC
Rt18s	Forward	GAGCTGGAATTACCGCGGCT
	Reverse	AAACGGCTACCACATCCAAG

## Data Availability

The original contributions presented in the study are included in the article/Supplementary Material, further inquiries can be directed to the corresponding author/s.

## Supplementary Materials

The following supporting information can be downloaded at: https://www.mdpi.com/article/10.3390/biom14101299/s1, Figure S1: Effects of Nutrient Deprivation on Yap Signaling.; Figure S2: Ischemic components with Glucose Supplementation.; Figure S3: Determination of Reference Genes for qPCR Analysis. The original western blot images can be found in Supplementary Materials.
