# Supplementary material for: Yap Is a Nutrient Sensor Sensitive to the Amino Acid L-Isoleucine and Regulates the Expression of Ctgf in Cardiomyocytes
Source: Biomolecules. 2024 Oct 14;14(10):1299. doi: 10.3390/biom14101299 (PMC11506509; doi:10.3390/biom14101299)
Supplement: Supplementary file 1 [file biomolecules-14-01299-s001.zip › biomolecules-3227860-Raw data of WB.pdf]

Raw Data Files:

Sup. Figure S2

p-Yap (S127)

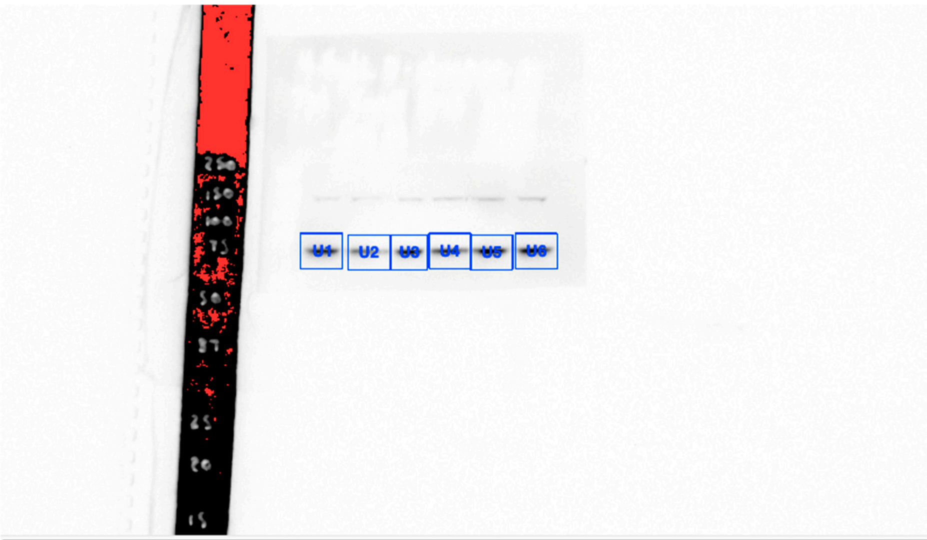

No.	Label	Type	Volume (Int)	Adj. Vol. (Int)	Mean Bkgd. (Int)	Abs. Quant.	Rel. Quant.	# of Pixels
1	U1	Unknown	3,124,432	2,236,876	1,101.2	N/A	N/A	806
2	U2	Unknown	1,901,792	1,130,846	956.5	N/A	N/A	806
3	U3	Unknown	2,410,564	1,738,635	957.2	N/A	N/A	702
4	U4	Unknown	2,691,308	1,687,742	1,245.1	N/A	N/A	806
5	U5	Unknown	3,422,396	2,333,668	1,350.8	N/A	N/A	806
6	U6	Unknown	3,548,824	2,332,584	1,509.0	N/A	N/A	806

P-Yap (S397)

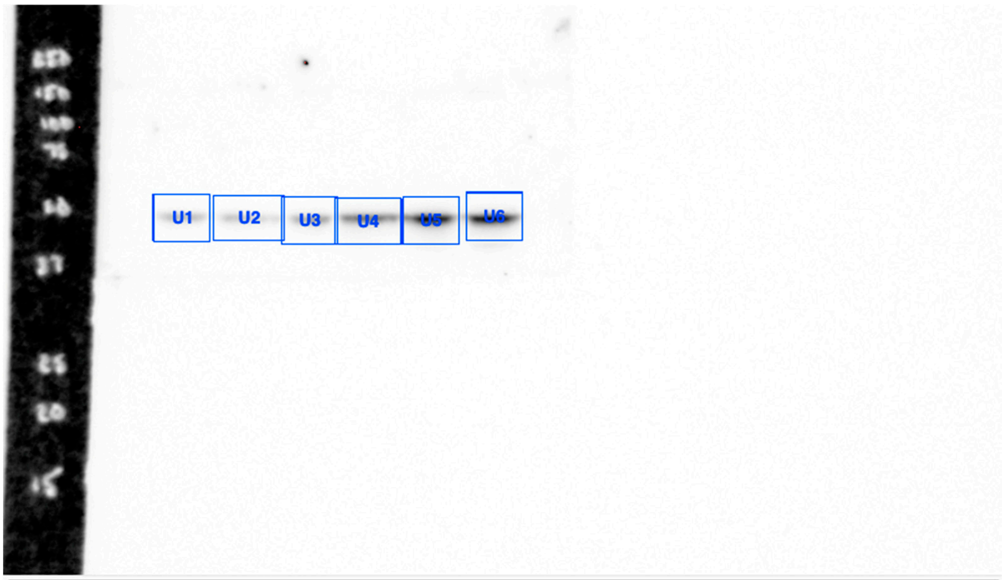

No.	Label	Type	Volume (Int)	Adj. Vol. (Int)	Mean Bkgd. (Int)	Abs. Quant.	Rel. Quant.	# of Pixels
1	U1	Unknown	2,698,288	2,273,891	329.8	N/A	N/A	1287
2	U2	Unknown	2,733,360	2,158,993	378.1	N/A	N/A	1519
3	U3	Unknown	3,641,496	2,534,572	860.1	N/A	N/A	1287
4	U4	Unknown	7,750,008	5,597,429	1,462.3	N/A	N/A	1472
5	U5	Unknown	10,885,288	8,315,566	1,996.7	N/A	N/A	1287
6	U6	Unknown	10,875,184	8,909,552	1,527.3	N/A	N/A	1287

Sup. Figure S2

Memcode for D-glucose

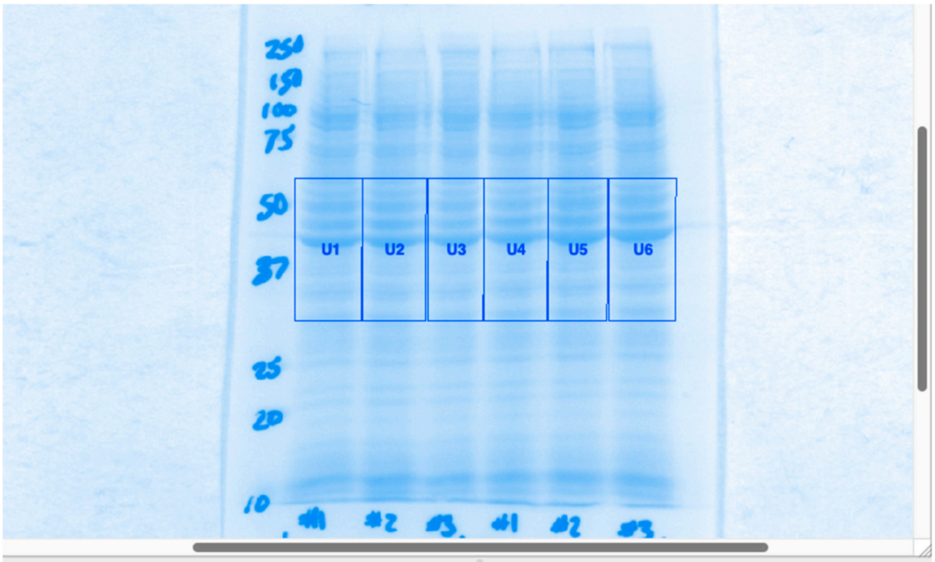

No.	Label	Type	Volume (Int)	Adj. Vol. (Int)	Mean Bkgd. (Int)	Abs. Quant.	Rel. Quant.	# of Pixels
1	U1	Unknown	300,881,121	33,351,175	29,610.4	N/A	N/A	9035
2	U2	Unknown	290,373,510	23,584,471	30,957.2	N/A	N/A	8618
3	U3	Unknown	246,393,179	20,157,814	29,592.6	N/A	N/A	7645
4	U4	Unknown	283,418,842	24,852,563	30,003.0	N/A	N/A	8618
5	U5	Unknown	273,969,402	28,863,090	30,402.7	N/A	N/A	8062
6	U6	Unknown	293,940,941	46,774,393	27,356.6	N/A	N/A	9035

Yap

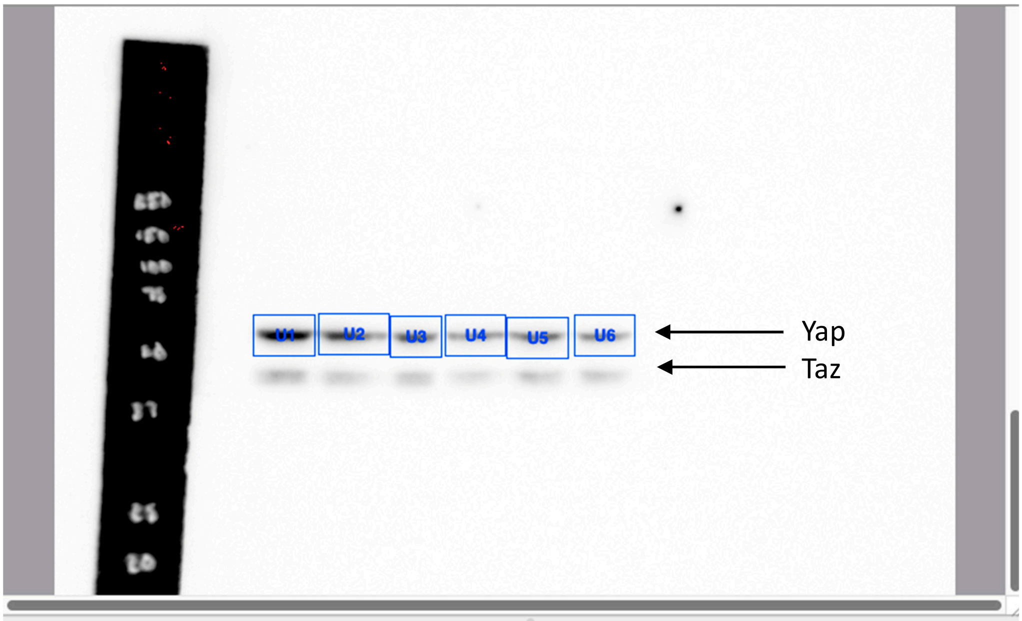

No.	Label	Type	Volume (Int)	Adj. Vol. (Int)	Mean Bkgd. (Int)	Abs. Quant.	Rel. Quant.	# of Pixels
1	U1	Unknown	12,375,796	10,431,477	1,292.8	N/A	N/A	1504
2	U2	Unknown	9,219,216	7,140,122	1,181.3	N/A	N/A	1760
3	U3	Unknown	5,781,384	4,704,993	862.5	N/A	N/A	1248
4	U4	Unknown	4,970,736	3,615,428	901.1	N/A	N/A	1504
5	U5	Unknown	5,978,644	4,703,623	847.8	N/A	N/A	1504
6	U6	Unknown	4,464,140	3,814,783	431.8	N/A	N/A	1504

Sup. Figure S1

Crop line (Another experiment on the right side that is not used in this paper)

P-Yap (S397)

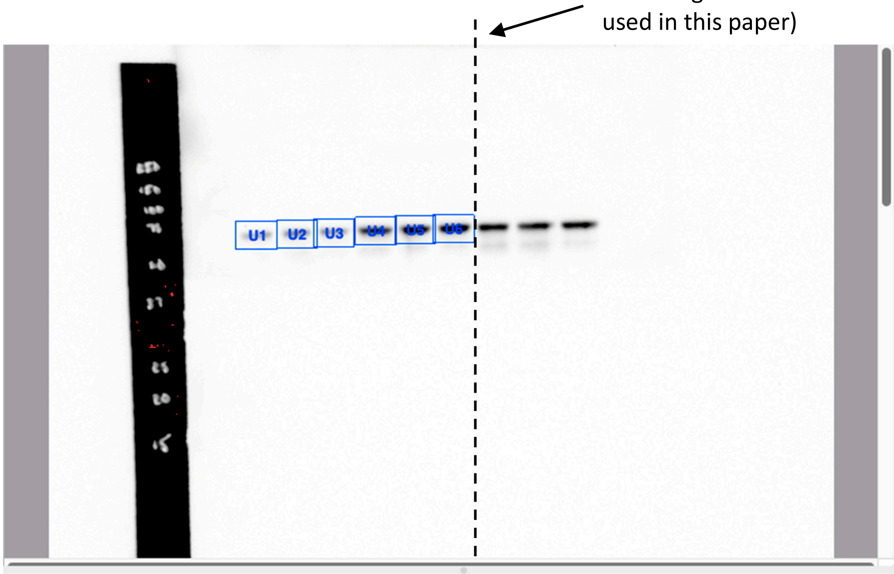

No.	Label	Type	Volume (Int)	Adj. Vol. (Int)	Mean Bkgd. (Int)	Abs. Quant.	Rel. Quant.	# of Pixels
1	U1	Unknown	1,759,256	1,459,685	332.9	N/A	N/A	900
2	U2	Unknown	2,375,172	1,798,201	641.1	N/A	N/A	900
3	U3	Unknown	2,276,248	1,665,419	678.7	N/A	N/A	900
4	U4	Unknown	5,591,980	4,522,494	1,188.3	N/A	N/A	900
5	U5	Unknown	6,440,152	5,246,981	1,325.7	N/A	N/A	900
6	U6	Unknown	6,948,560	5,992,846	1,061.9	N/A	N/A	900

Crop line (Another experiment on the right side that is not used in this paper)

P-Yap (S127)

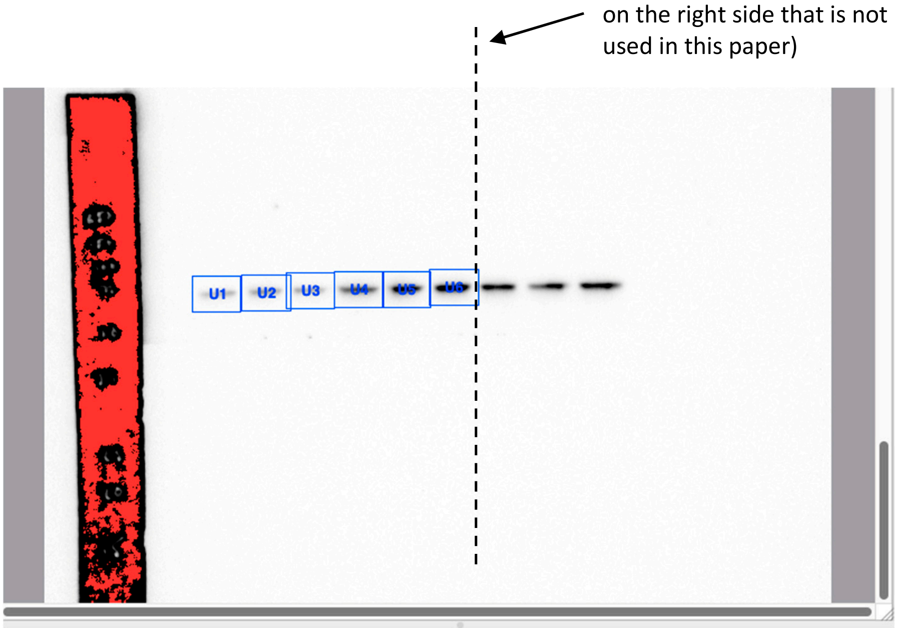

No.	Label	Type	Volume (Int)	Adj. Vol. (Int)	Mean Bkgd. (Int)	Abs. Quant.	Rel. Quant.	# of Pixels
1	U1	Unknown	1,016,564	801,908	156.0	N/A	N/A	1376
2	U2	Unknown	1,364,240	1,051,191	227.5	N/A	N/A	1376
3	U3	Unknown	1,164,156	834,810	239.4	N/A	N/A	1376
4	U4	Unknown	3,025,780	2,522,950	365.4	N/A	N/A	1376
5	U5	Unknown	3,878,324	3,280,389	434.5	N/A	N/A	1376
6	U6	Unknown	4,986,548	4,369,921	448.1	N/A	N/A	1376

Sup. Figure S1

Crop line (Another experiment on the right side that is not used in this paper)

Memcode Nutrient Deprivation

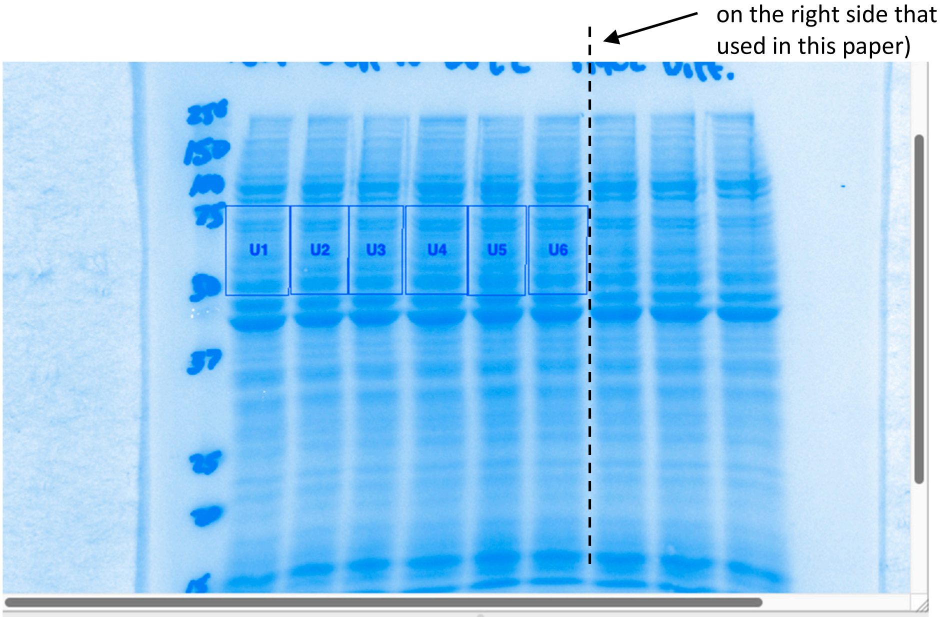

No.	Label	Type	Volume (Int)	Adj. Vol. (Int)	Mean Bkgd. (Int)	Abs. Quant.	Rel. Quant.	# of Pixels
1	U1	Unknown	306,054,782	42,529,761	27,850.9	N/A	N/A	9462
2	U2	Unknown	279,175,736	32,600,158	29,229.0	N/A	N/A	8436
3	U3	Unknown	268,830,264	27,818,476	30,202.0	N/A	N/A	7980
4	U4	Unknown	319,593,372	40,304,309	29,876.9	N/A	N/A	9348
5	U5	Unknown	296,287,008	37,635,021	29,853.6	N/A	N/A	8664
6	U6	Unknown	291,213,760	35,505,367	29,513.9	N/A	N/A	8664

Crop line (Another experiment on the right side that is not used in this paper)

Yap

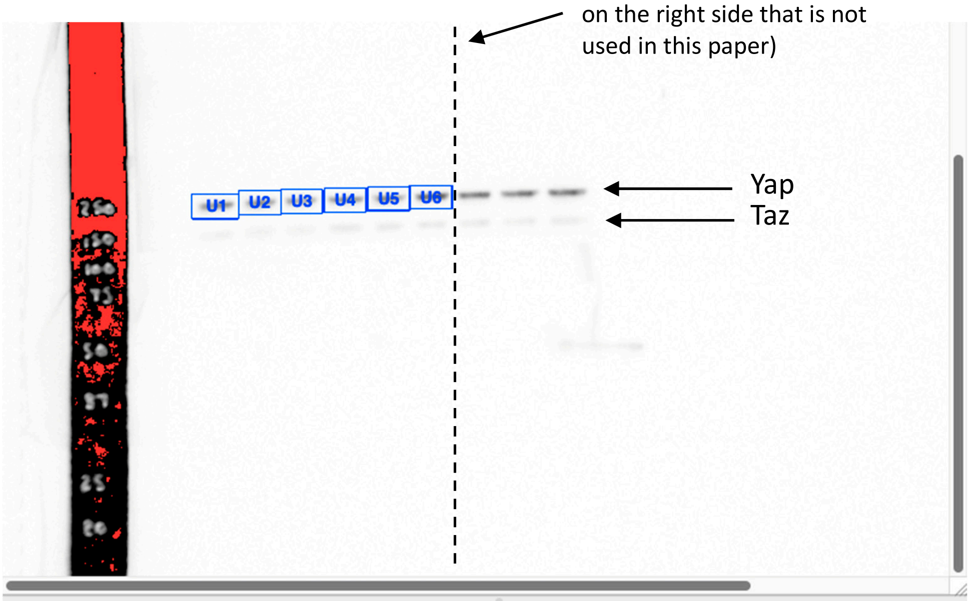

No.	Label	Type	Volume (Int)	Adj. Vol. (Int)	Mean Bkgd. (Int)	Abs. Quant.	Rel. Quant.	# of Pixels
1	U1	Unknown	2,079,296	1,858,687	371.4	N/A	N/A	594
2	U2	Unknown	1,811,496	1,551,063	510.7	N/A	N/A	510
3	U3	Unknown	1,566,020	1,321,657	479.1	N/A	N/A	510
4	U4	Unknown	1,853,288	1,651,932	394.8	N/A	N/A	510
5	U5	Unknown	1,896,520	1,666,604	450.8	N/A	N/A	510
6	U6	Unknown	2,429,536	2,259,945	332.5	N/A	N/A	510

Sup. Figure S1

Crop line (Another experiment on the left side that is not used in this paper)

P-Yap (S397)

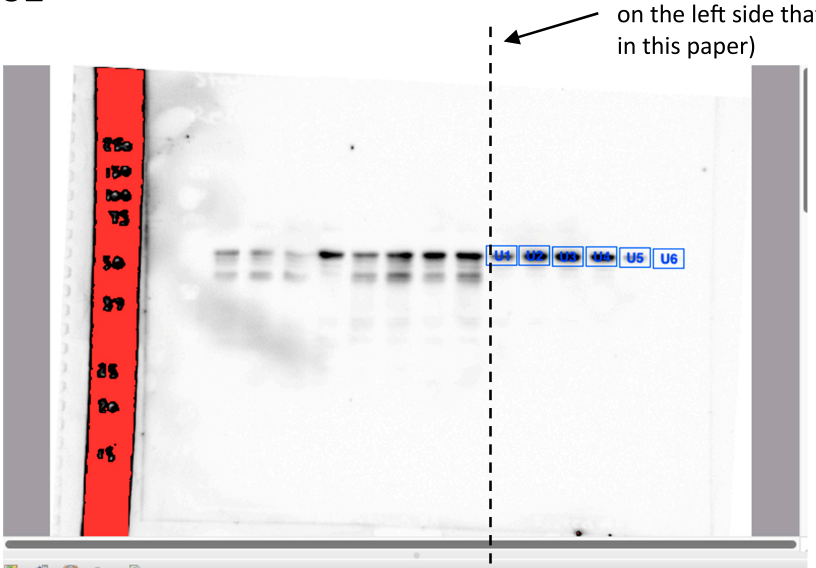

No.	Label	Type	Volume (Int)	Adj. Vol. (Int)	Mean Bkgd. (Int)	Abs. Quant.	Rel. Quant.	# of Pixels
1	U1	Unknown	4,049,954	3,102,793	1,527.7	N/A	N/A	620
2	U2	Unknown	8,267,708	6,702,003	2,525.3	N/A	N/A	620
3	U3	Unknown	9,333,320	7,890,106	2,327.8	N/A	N/A	620
4	U4	Unknown	7,066,288	6,360,564	1,138.3	N/A	N/A	620
5	U5	Unknown	1,729,375	1,561,501	270.8	N/A	N/A	620
6	U6	Unknown	354,108	264,799	144.0	N/A	N/A	620

Crop line (Another experiment on the left side that is not used in this paper)

P-Yap (S127)

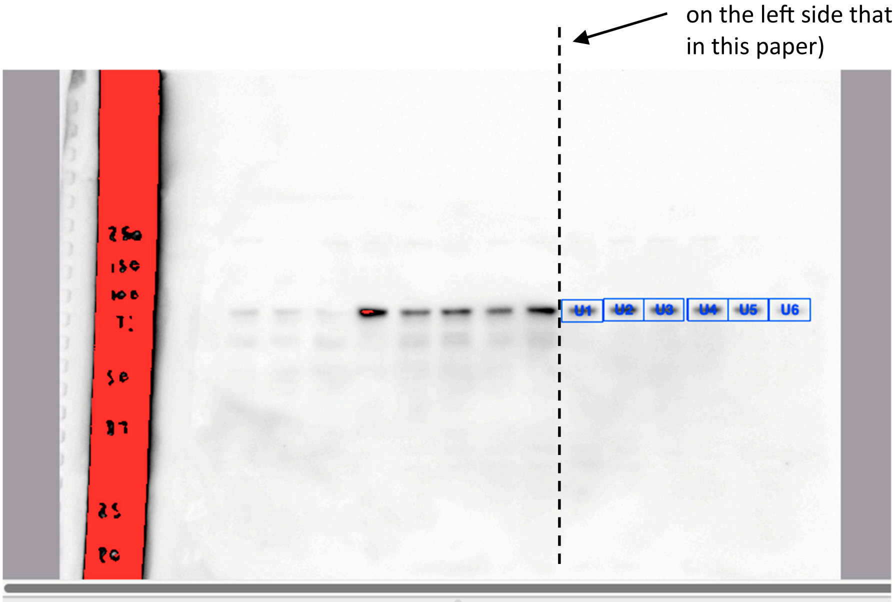

No.	Label	Type	Volume (Int)	Adj. Vol. (Int)	Mean Bkgd. (Int)	Abs. Quant.	Rel. Quant.	# of Pixels
1	U1	Unknown	5,016,264	3,433,083	2,198.9	N/A	N/A	720
2	U2	Unknown	5,259,944	3,943,983	1,827.7	N/A	N/A	720
3	U3	Unknown	5,168,124	3,566,596	2,224.3	N/A	N/A	720
4	U4	Unknown	5,238,692	3,647,641	2,209.8	N/A	N/A	720
5	U5	Unknown	3,884,116	2,794,607	1,513.2	N/A	N/A	720
6	U6	Unknown	2,199,852	1,371,579	1,150.4	N/A	N/A	720

Sup. Figure S1

Refeed Memcode

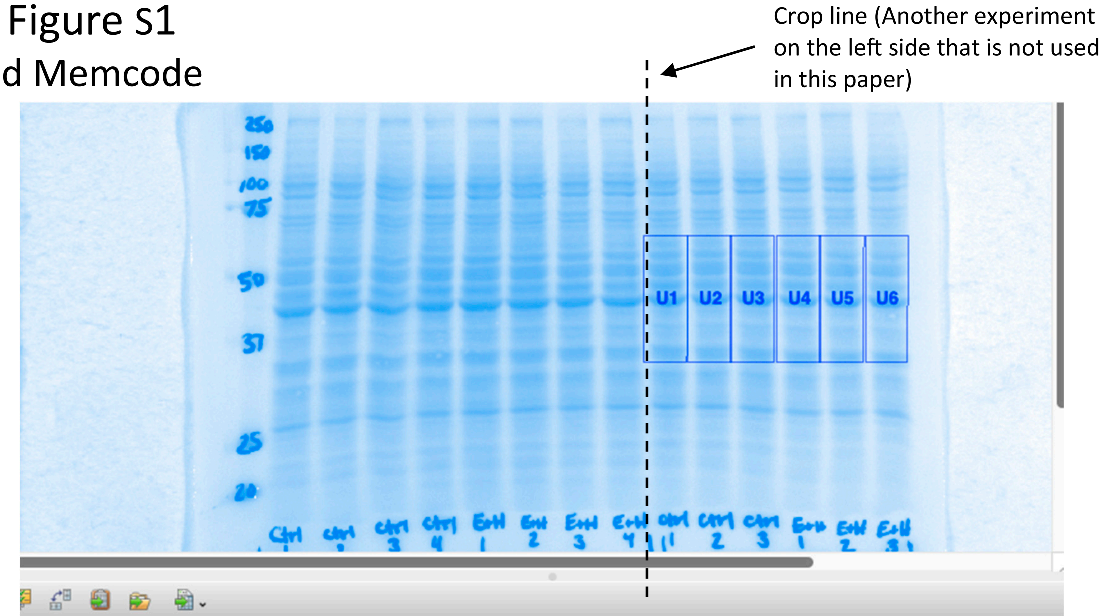

No.	Label	Type	Volume (Int)	Adj. Vol. (Int)	Mean Bkgd. (Int)	Abs. Quant.	Rel. Quant.	# of Pixels
1	U1	Unknown	319,730,305	64,631,922	27,456.5	N/A	N/A	9291
2	U2	Unknown	309,627,021	61,887,277	26,664.5	N/A	N/A	9291
3	U3	Unknown	311,209,373	66,186,954	26,372.0	N/A	N/A	9291
4	U4	Unknown	298,240,581	59,802,467	25,663.3	N/A	N/A	9291
5	U5	Unknown	292,364,353	59,784,843	25,032.8	N/A	N/A	9291
6	U6	Unknown	280,162,263	62,120,586	24,321.4	N/A	N/A	8965

Yap

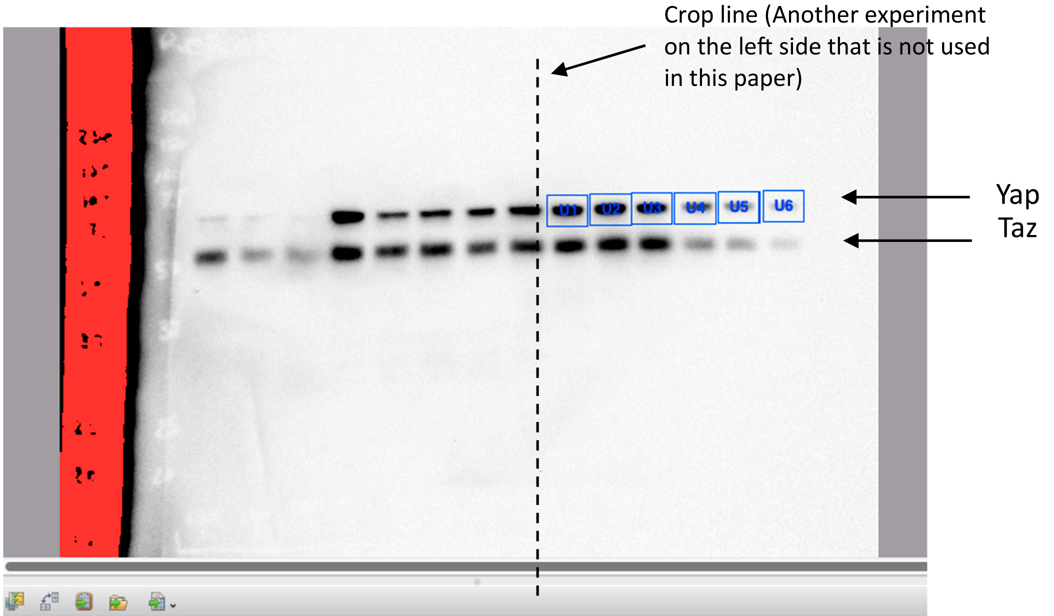

No.	Label	Type	Volume (Int)	Adj. Vol. (Int)	Mean Bkgd. (Int)	Abs. Quant.	Rel. Quant.	# of Pixels
1	U1	Unknown	4,643,348	3,531,792	1,176.3	N/A	N/A	945
2	U2	Unknown	5,459,028	4,438,812	1,079.6	N/A	N/A	945
3	U3	Unknown	3,727,048	3,029,579	738.1	N/A	N/A	945
4	U4	Unknown	1,737,648	1,314,672	447.6	N/A	N/A	945
5	U5	Unknown	1,139,560	822,926	335.1	N/A	N/A	945
6	U6	Unknown	679,276	408,445	286.6	N/A	N/A	945

Figure 3

Yap

Isoleucine and Threonine blot

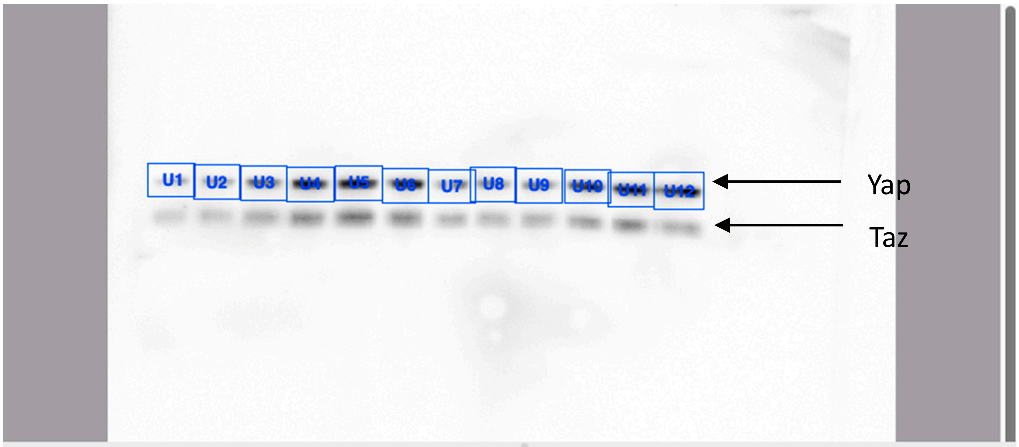

No.	Label	Type	Volume (Int)	Adj. Vol. (Int)	Mean Bkgd. (Int)	Abs. Quant.	Rel. Quant.	# of Pixels
1	U1	Unknown	3,033,576	1,627,703	1,143.0	N/A	N/A	1230
2	U2	Unknown	4,299,256	2,492,976	1,468.5	N/A	N/A	1230
3	U3	Unknown	7,003,696	4,670,403	1,897.0	N/A	N/A	1230
4	U4	Unknown	10,842,888	7,412,469	2,789.0	N/A	N/A	1230
5	U5	Unknown	11,789,392	8,851,916	2,388.2	N/A	N/A	1230
6	U6	Unknown	9,956,556	8,155,533	1,464.2	N/A	N/A	1230
7	U7	Unknown	5,071,368	2,944,850	1,728.9	N/A	N/A	1230
8	U8	Unknown	5,309,184	3,516,248	1,457.7	N/A	N/A	1230
9	U9	Unknown	5,266,476	3,738,850	1,242.0	N/A	N/A	1230
10	U10	Unknown	8,780,776	6,784,200	1,623.2	N/A	N/A	1230
11	U11	Unknown	11,245,516	8,210,280	2,467.7	N/A	N/A	1230
12	U12	Unknown	12,514,680	9,261,921	2,258.9	N/A	N/A	1440

Valine blot

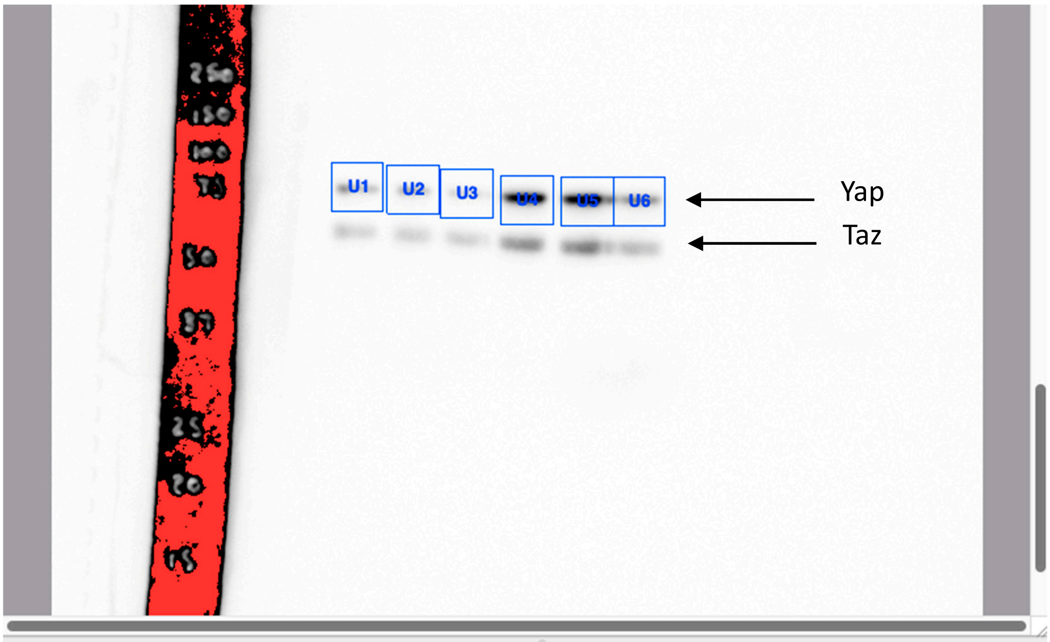

No.	Label	Type	Volume (Int)	Adj. Vol. (Int)	Mean Bkgd. (Int)	Abs. Quant.	Rel. Quant.	# of Pixels
1	U1	Unknown	2,433,776	1,953,061	342.4	N/A	N/A	1404
2	U2	Unknown	1,960,232	1,546,216	294.9	N/A	N/A	1404
3	U3	Unknown	1,399,552	894,440	359.8	N/A	N/A	1404
4	U4	Unknown	8,784,748	7,428,557	965.9	N/A	N/A	1404
5	U5	Unknown	10,002,328	7,777,225	1,584.8	N/A	N/A	1404
6	U6	Unknown	3,972,752	2,426,857	1,101.1	N/A	N/A	1404

Figure 3

p-Yap (S127)

Isoleucine and Threonine

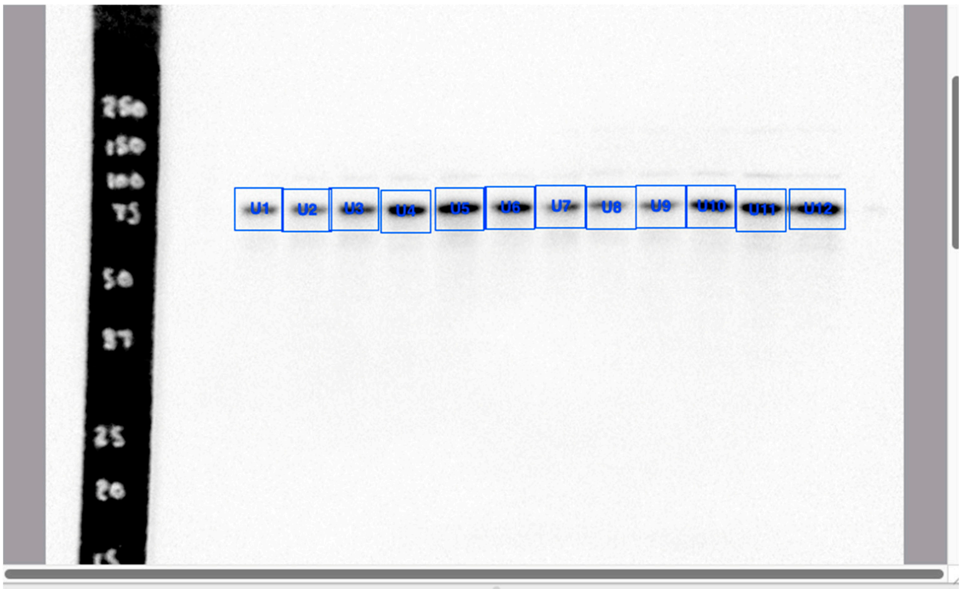

No.	Label	Type	Volume (Int)	Adj. Vol. (Int)	Mean Bkgd. (Int)	Abs. Quant.	Rel. Quant.	# of Pixels
1	U1	Unknown	1,258,524	985,615	194.9	N/A	N/A	1400
2	U2	Unknown	1,392,024	986,606	289.6	N/A	N/A	1400
3	U3	Unknown	1,968,076	1,377,276	422.0	N/A	N/A	1400
4	U4	Unknown	2,919,940	2,301,031	442.1	N/A	N/A	1400
5	U5	Unknown	3,373,784	2,605,639	548.7	N/A	N/A	1400
6	U6	Unknown	2,707,124	2,180,288	376.3	N/A	N/A	1400

Valine blots

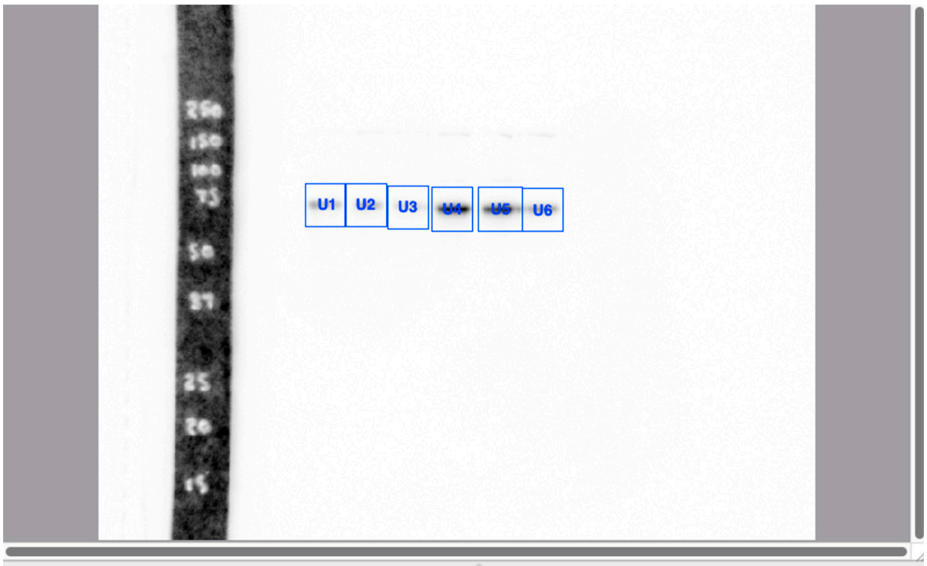

No.	Label	Type	Volume (Int)	Adj. Vol. (Int)	Mean Bkgd. (Int)	Abs. Quant.	Rel. Quant.	# of Pixels
1	U1	Unknown	3,650,328	2,818,488	495.1	N/A	N/A	1680
2	U2	Unknown	3,330,360	2,558,120	459.7	N/A	N/A	1680
3	U3	Unknown	2,552,600	1,498,440	627.5	N/A	N/A	1680
4	U4	Unknown	12,177,120	10,636,400	917.1	N/A	N/A	1680
5	U5	Unknown	11,222,408	8,971,585	1,276.0	N/A	N/A	1764
6	U6	Unknown	4,806,564	3,097,404	1,017.4	N/A	N/A	1680

Figure 3

p-Yap (S397)

Isoleucine and Threonine

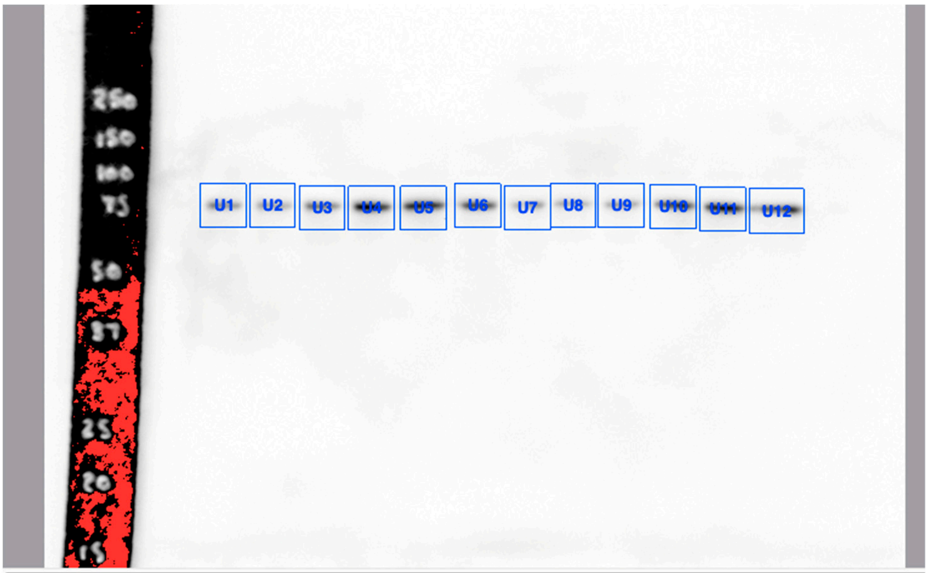

No.	Label	Type	Volume (Int)	Adj. Vol. (Int)	Mean Bkgd. (Int)	Abs. Quant.	Rel. Quant.	# of Pixe
1	U1	Unknown	5,293,384	3,023,144	1,753.1	N/A	N/A	[illegible]
2	U2	Unknown	4,220,024	2,595,709	1,254.3	N/A	N/A	[illegible]
3	U3	Unknown	5,545,748	3,649,133	1,464.6	N/A	N/A	[illegible]
4	U4	Unknown	9,384,320	7,121,325	1,747.5	N/A	N/A	[illegible]
5	U5	Unknown	9,736,448	7,291,208	1,888.2	N/A	N/A	[illegible]
6	U6	Unknown	6,894,144	5,288,869	1,239.6	N/A	N/A	[illegible]

Valine Blot

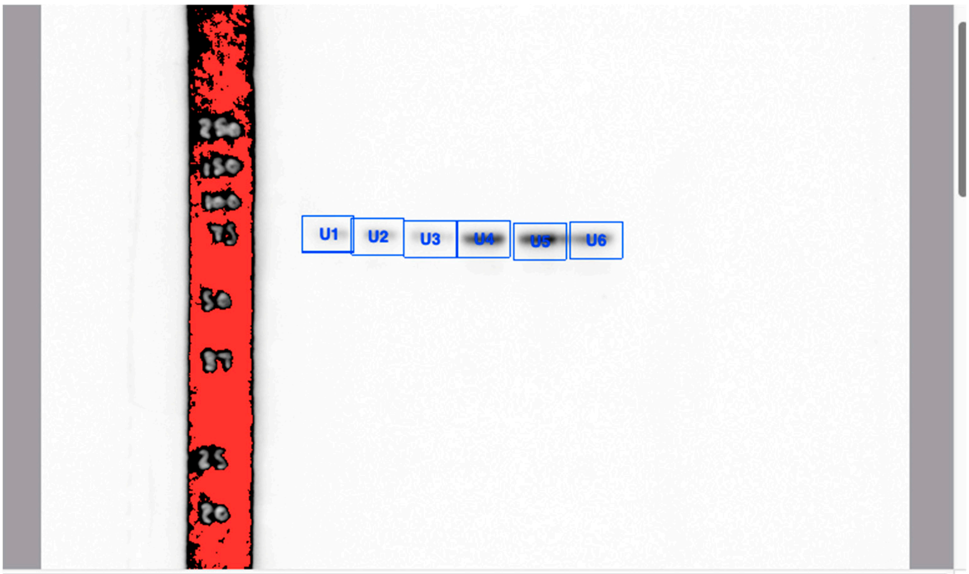

No.	Label	Type	Volume (Int)	Adj. Vol. (Int)	Mean Bkgd. (Int)	Abs. Quant.	Rel. Quant.	# of Pixels
1	U1	Unknown	2,308,036	1,651,217	539.3	N/A	N/A	1218
2	U2	Unknown	2,935,156	2,371,639	462.7	N/A	N/A	1218
3	U3	Unknown	2,657,948	1,856,504	658.0	N/A	N/A	1218
4	U4	Unknown	8,994,688	7,840,758	947.4	N/A	N/A	1218
5	U5	Unknown	10,952,520	8,341,261	2,143.9	N/A	N/A	1218
6	U6	Unknown	5,914,516	4,144,812	1,453.0	N/A	N/A	1218

Figure 3

Memcodes

Crop lines, only one control included in the figure

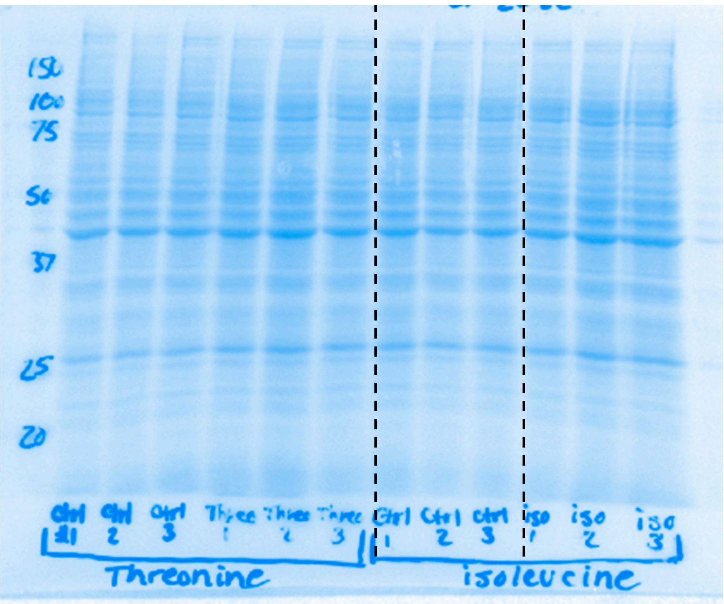

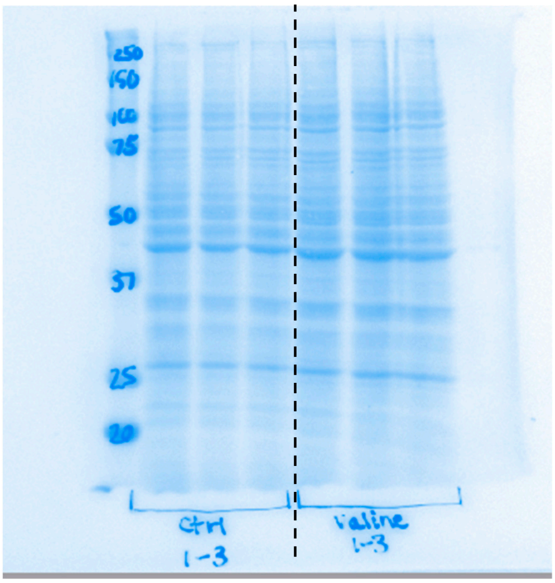

Figures 2&4

P-Yap (S397)

Nuclear

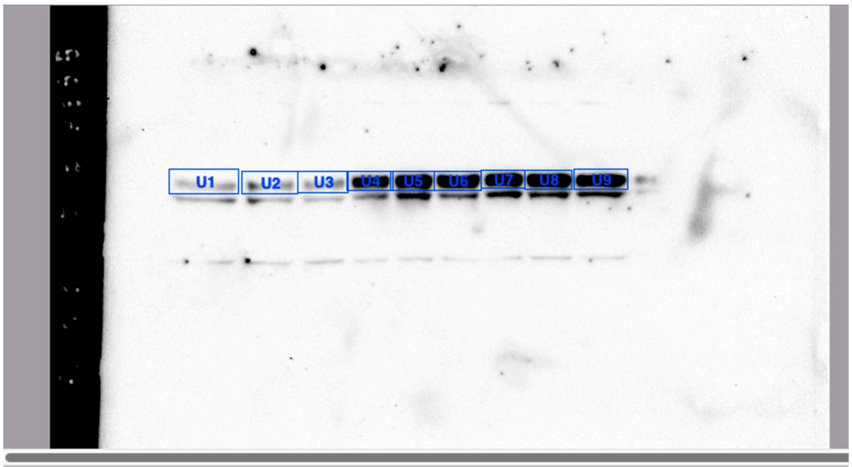

No.	Label	Type	Volume (Int)	Adj. Vol. (Int)	Mean Bkgd. (Int)	Abs. Quant.	Rel. Quant.	# of Pi
1	U1	Unknown	1,362,516	709,128	479.0	N/A	N/A	
2	U2	Unknown	1,511,060	974,782	536.3	N/A	N/A	
3	U3	Unknown	1,300,132	766,249	624.4	N/A	N/A	
4	U4	Unknown	5,084,560	3,616,608	2,106.1	N/A	N/A	
5	U5	Unknown	8,398,860	6,235,971	3,262.3	N/A	N/A	
6	U6	Unknown	8,400,304	6,449,703	2,731.9	N/A	N/A	
7	U7	Unknown	7,829,748	5,849,829	3,172.9	N/A	N/A	
8	U8	Unknown	10,930,528	8,579,150	3,293.2	N/A	N/A	
9	U9	Unknown	9,748,296	7,418,723	2,696.3	N/A	N/A	

Cytoplasmic

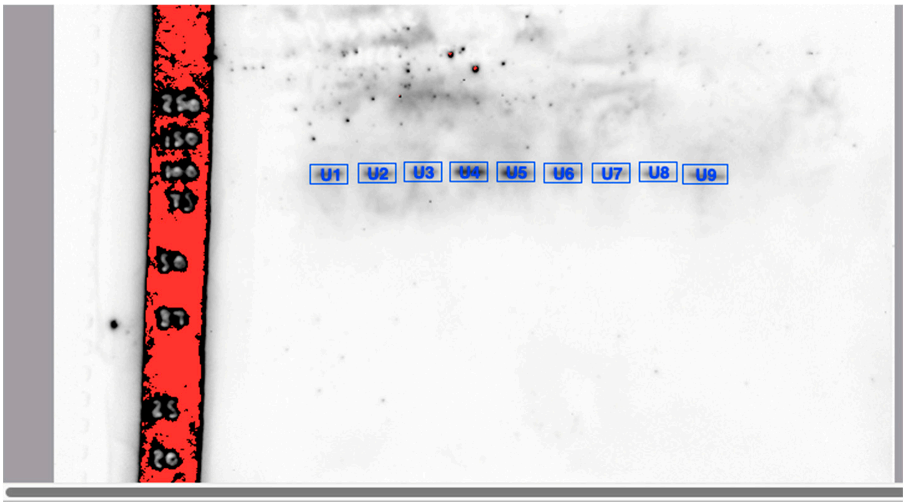

No.	Label	Type	Volume (Int)	Adj. Vol. (Int)	Mean Bkgd. (Int)	Abs. Quant.	Rel. Quant.	# of I
1	U1	Unknown	4,617,252	2,156,930	4,960.3	N/A	N/A	
2	U2	Unknown	4,681,872	2,045,764	5,314.7	N/A	N/A	
3	U3	Unknown	4,235,616	1,747,923	5,015.5	N/A	N/A	
4	U4	Unknown	6,466,872	3,415,359	6,152.2	N/A	N/A	
5	U5	Unknown	5,735,576	2,649,039	6,222.9	N/A	N/A	
6	U6	Unknown	4,282,424	2,047,752	4,505.4	N/A	N/A	
7	U7	Unknown	3,369,544	1,621,620	3,524.0	N/A	N/A	
8	U8	Unknown	3,078,240	1,522,339	3,136.9	N/A	N/A	
9	U9	Unknown	4,814,452	2,291,628	4,261.5	N/A	N/A	

Figures 2&4

P-Yap (S127)

Nuclear

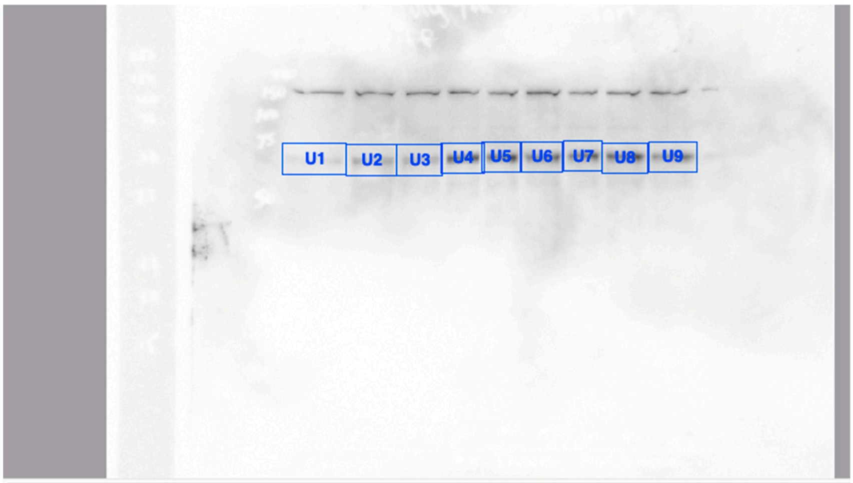

No.	Label	Type	Volume (Int)	Adj. Vol. (Int)	Mean Bkgd. (Int)	Abs. Quant.	Rel. Quant.
1	U1	Unknown	9,980,564	1,146,236	4,913.4	N/A	N/A
2	U2	Unknown	12,965,596	2,810,868	7,295.1	N/A	N/A
3	U3	Unknown	12,261,628	2,191,844	7,891.7	N/A	N/A
4	U4	Unknown	13,960,832	4,944,348	7,583.3	N/A	N/A
5	U5	Unknown	13,722,128	5,150,531	7,988.4	N/A	N/A
6	U6	Unknown	14,968,292	5,312,485	8,324.0	N/A	N/A
7	U7	Unknown	14,040,396	4,934,571	8,486.3	N/A	N/A
8	U8	Unknown	17,087,932	6,500,498	8,490.3	N/A	N/A
9	U9	Unknown	15,800,960	5,609,477	7,639.8	N/A	N/A

Cytoplasmic

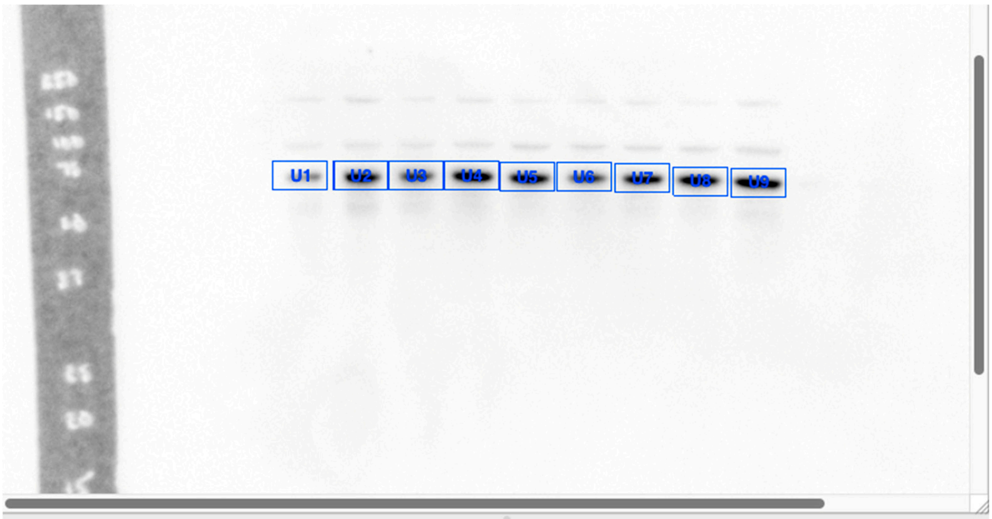

No.	Label	Type	Volume (Int)	Adj. Vol. (Int)	Mean Bkgd. (Int)	Abs. Quant.	Rel. Quant.	# of Pixels
1	U1	Unknown	1,647,440	1,243,905	531.0	N/A	N/A	760
2	U2	Unknown	4,943,540	3,934,235	1,328.0	N/A	N/A	760
3	U3	Unknown	3,845,708	2,658,056	1,562.7	N/A	N/A	760
4	U4	Unknown	5,968,548	4,783,936	1,558.7	N/A	N/A	760
5	U5	Unknown	5,230,684	4,176,437	1,387.2	N/A	N/A	760
6	U6	Unknown	3,596,568	2,566,540	1,355.3	N/A	N/A	760
7	U7	Unknown	4,666,876	3,728,175	1,235.1	N/A	N/A	760
8	U8	Unknown	5,333,136	4,441,707	1,172.9	N/A	N/A	760
9	U9	Unknown	5,919,104	4,992,157	1,219.7	N/A	N/A	760

Figures 2 & 4

Lamin a/c

Nuclear fraction (300s exposure)

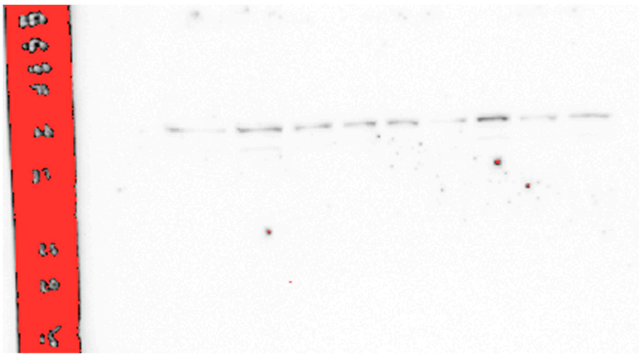

Cytoplasmic fraction (300s exposure)

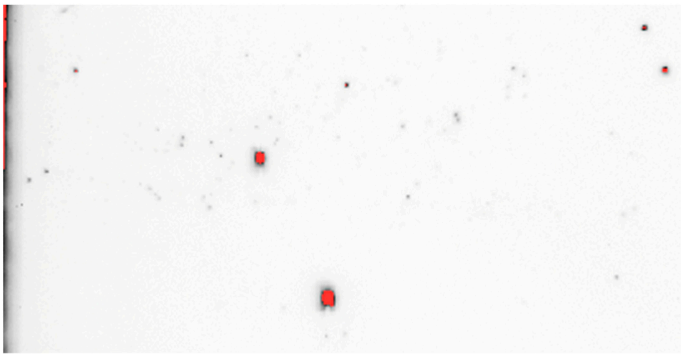

Gapdh

Nuclear fraction (300s exposure)

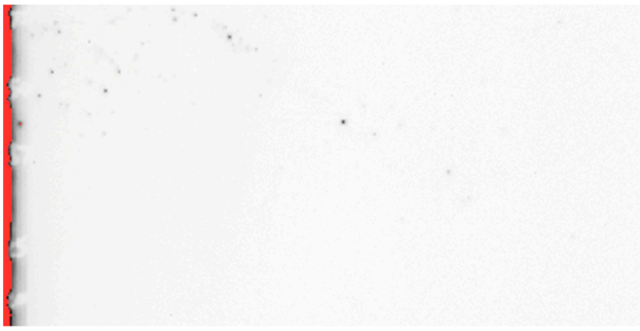

Cytoplasmic fraction (300s exposure)

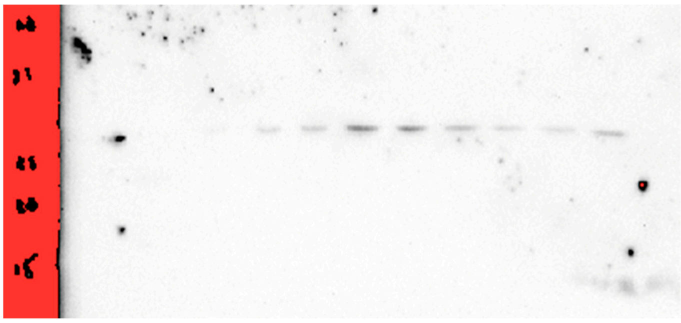

Figures 2 &4 representative blots

Memcode: Nuclear Fraction

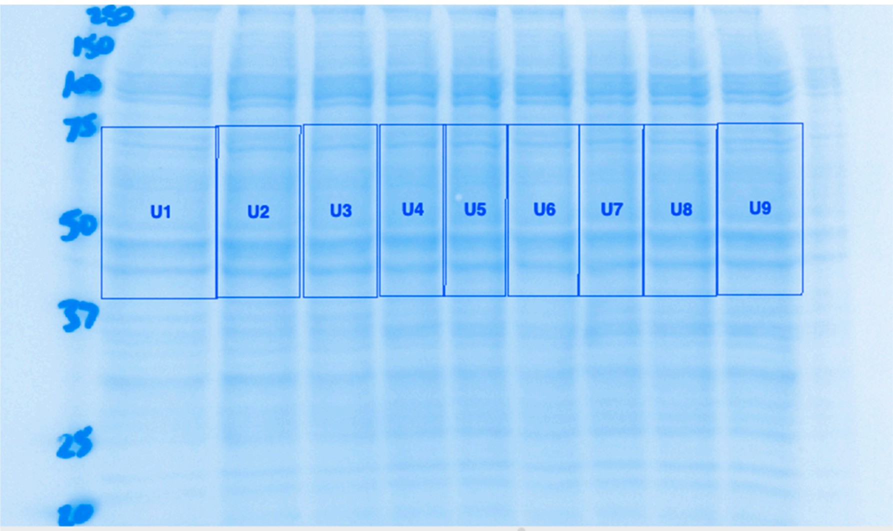

No.	Label	Type	Volume (Int)	Adj. Vol. (Int)	Mean Bkgd. (Int)	Abs. Quant.	Rel. Quant.
1	U1	Unknown	725,828,124	68,648,725	25,210.2	N/A	N/A
2	U2	Unknown	583,976,156	101,398,492	25,647.2	N/A	N/A
3	U3	Unknown	512,803,484	75,677,521	26,238.1	N/A	N/A
4	U4	Unknown	472,706,968	63,714,998	27,456.5	N/A	N/A
5	U5	Unknown	454,325,248	73,435,782	26,990.5	N/A	N/A
6	U6	Unknown	477,148,052	62,039,987	26,146.9	N/A	N/A
7	U7	Unknown	438,586,656	67,906,766	25,907.2	N/A	N/A
8	U8	Unknown	495,877,360	76,144,608	25,801.1	N/A	N/A
9	U9	Unknown	560,111,308	93,088,645	24,314.0	N/A	N/A

Memcode: Cytoplasm Fraction

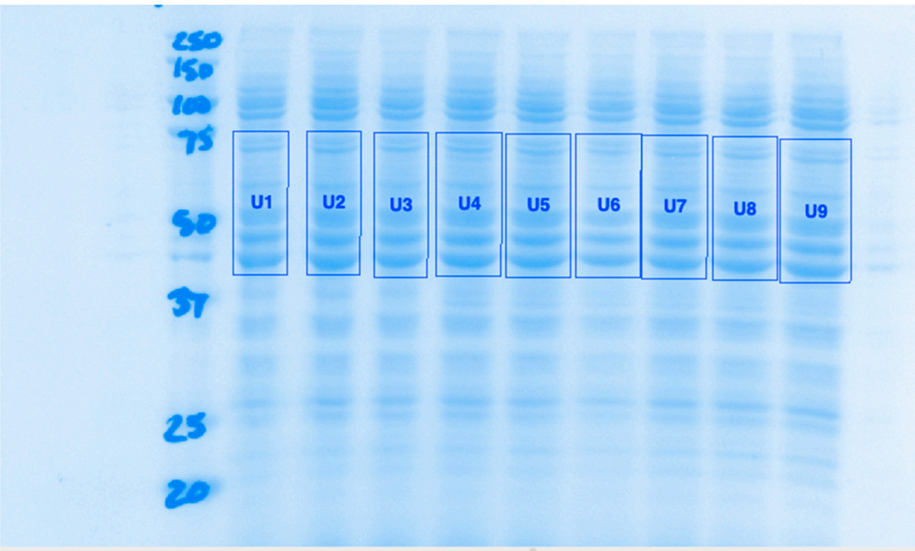

No.	Label	Type	Volume (Int)	Adj. Vol. (Int)	Mean Bkgd. (Int)	Abs. Quant.	Rel. Quant.
1	U1	Unknown	298,610,940	62,598,756	24,895.8	N/A	N/A
2	U2	Unknown	316,152,400	68,365,625	26,137.8	N/A	N/A
3	U3	Unknown	302,869,588	58,221,210	25,806.8	N/A	N/A
4	U4	Unknown	367,289,972	71,269,486	26,021.5	N/A	N/A
5	U5	Unknown	363,265,580	72,027,231	25,601.1	N/A	N/A
6	U6	Unknown	319,921,528	44,233,681	24,234.2	N/A	N/A
7	U7	Unknown	348,054,796	66,585,587	24,742.4	N/A	N/A
8	U8	Unknown	338,843,556	61,195,506	24,406.5	N/A	N/A
9	U9	Unknown	367,551,708	80,498,407	23,292.2	N/A	N/A

Figures 2&4

Yap

Nuclear

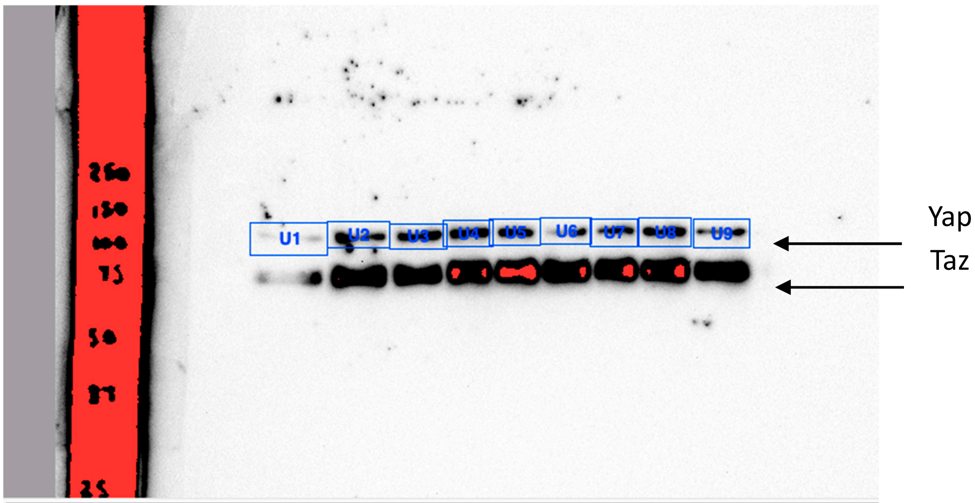

Yap

Taz

No.	Label	Type	Volume (Int)	Adj. Vol. (Int)	Mean Bkgd. (Int)	Abs. Quant.	Rel. Quant.
1	U1	Unknown	1,003,308	249,143	467.8	N/A	N/A
2	U2	Unknown	4,777,919	2,548,956	2,122.8	N/A	N/A
3	U3	Unknown	4,302,680	3,324,422	1,063.3	N/A	N/A
4	U4	Unknown	4,481,652	3,484,453	1,158.2	N/A	N/A
5	U5	Unknown	3,460,908	2,669,864	918.8	N/A	N/A
6	U6	Unknown	1,748,608	1,186,940	652.3	N/A	N/A
7	U7	Unknown	2,617,164	1,892,864	866.4	N/A	N/A
8	U8	Unknown	4,746,812	3,886,697	890.4	N/A	N/A
9	U9	Unknown	2,474,012	1,838,640	613.9	N/A	N/A

Cytoplasmic

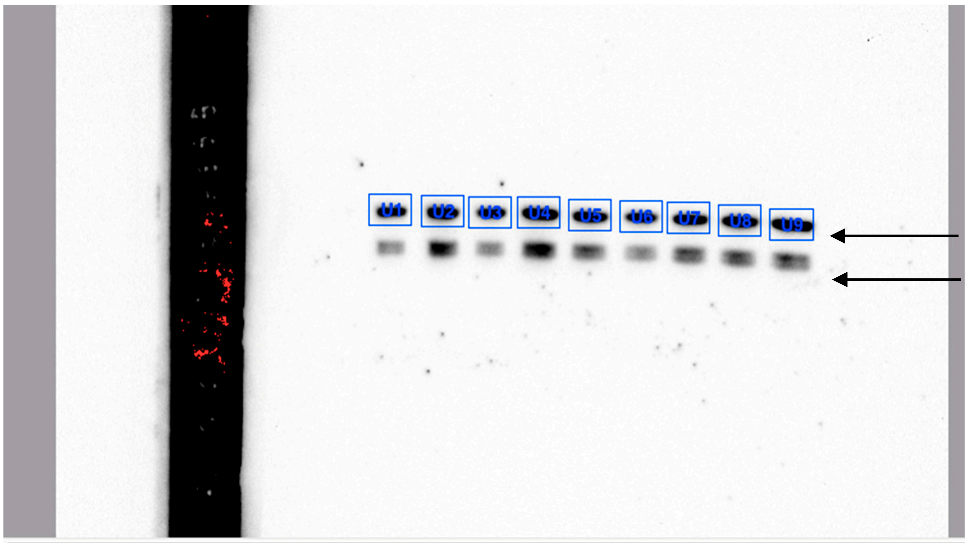

Yap

Taz

No.	Label	Type	Volume (Int)	Adj. Vol. (Int)	Mean Bkgd. (Int)	Abs. Quant.	Rel. Quant.	# of P
1	U1	Unknown	3,195,548	2,926,339	354.7	N/A	N/A	
2	U2	Unknown	4,909,316	4,446,117	610.3	N/A	N/A	
3	U3	Unknown	3,100,472	2,777,478	425.6	N/A	N/A	
4	U4	Unknown	6,022,656	5,443,199	763.4	N/A	N/A	
5	U5	Unknown	4,540,560	4,135,516	533.7	N/A	N/A	
6	U6	Unknown	3,376,044	3,099,585	364.2	N/A	N/A	
7	U7	Unknown	4,489,128	4,105,100	506.0	N/A	N/A	
8	U8	Unknown	4,982,340	4,508,933	623.7	N/A	N/A	
9	U9	Unknown	6,327,920	5,738,610	753.6	N/A	N/A	

Figure 1:

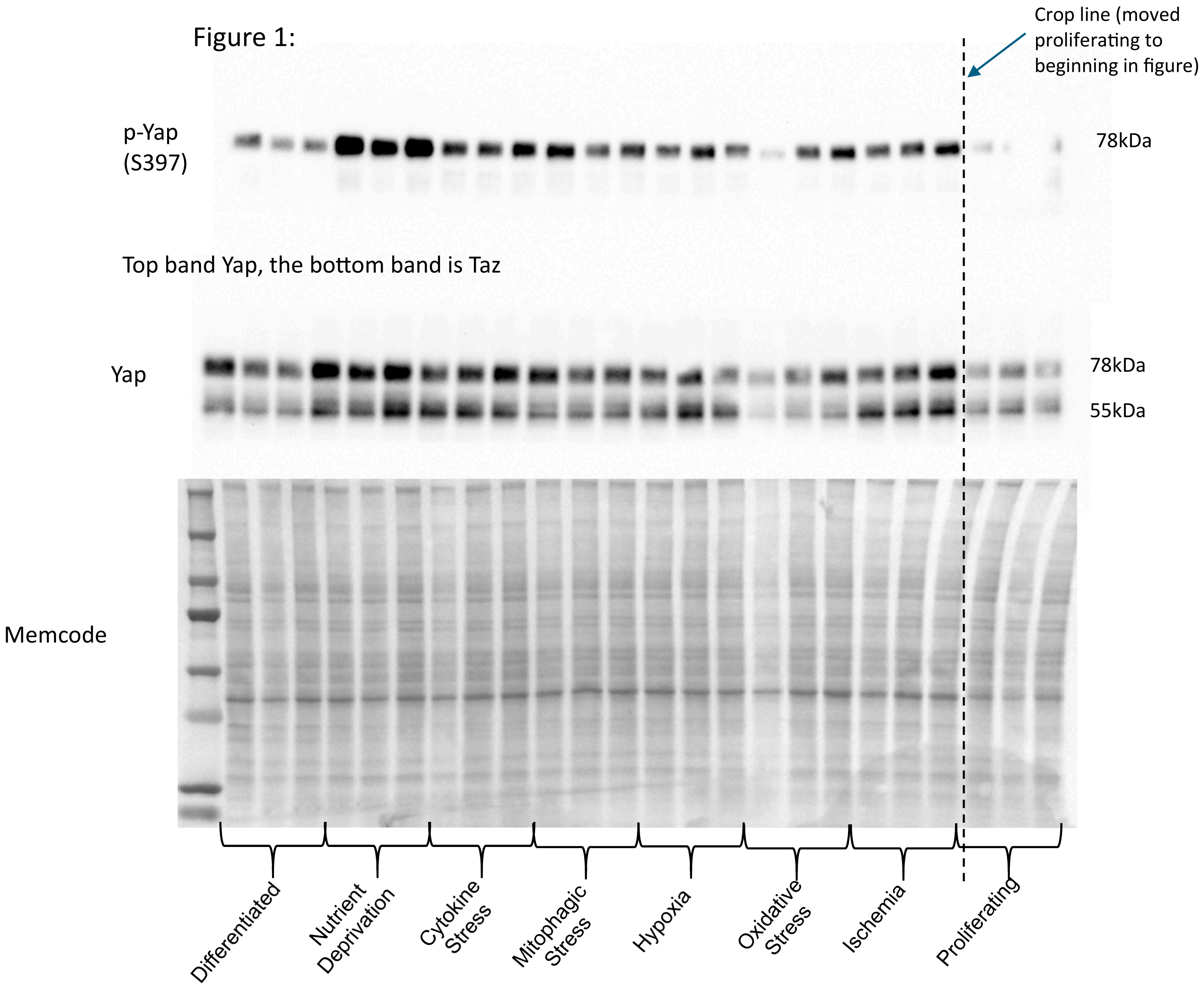

Lane	1	2	3	4	5	6	7	8	9	10	11	12	13	14	15
Memcode	Controls	Controls	Controls	Starvation	Starvation	Starvation	tnfa	tnfa	tnfa	FCCP	FCCP	FCCP	Hypoxia	Hypoxia	Hypoxia
	42735298.2	41852140.5	41849981.3	57887133.2	46262008.5	57292172.5	57731855.9	55423393.4	52241385.3	59410173.4	52073036	54131377.6	44956028.7	45897250.5	43657947.9
Yap	5,375,861.00	3,671,801.00	2,985,280.00	7,681,659.33	5,403,867.67	6,925,825.67	4,352,874.00	5,225,308.67	6,198,688.67	5,784,154.67	3,820,750.00	4,707,592.00	3,682,563.33	3,365,308.00	2,362,693.00
adjusted	12.5794395	8.77326931	7.13328873	13.2700635	11.6810053	12.0886072	7.53981304	9.42798402	11.8654753	9.73596665	7.33729065	8.69660483	8.1914783	7.33226492	5.41182789
normalized	1.32554684	0.92447516	0.75166372	1.39832071	1.23087516	1.27382583	0.79450085	0.99346512	1.25031352	1.02591851	0.77316024	0.91639672	0.86316947	0.77263066	0.57026637
p-Yap S397	1,676,185.22	871,589.04	1,126,877.74	7,704,259.48	4,436,497.74	7,318,740.35	2,395,634.09	2,179,499.30	2,972,341.04	3,145,130.43	1,624,202.26	2,284,417.74	1,607,779.48	2,329,198.43	1,509,520.17
	3.92224996	2.08254353	2.69266008	13.3091052	9.58993759	12.7744158	4.14958786	3.93245374	5.68962907	5.2939257	3.11908501	4.22013597	3.57633787	5.07481038	3.45760679
	1.35296653	0.71836617	0.92882376	4.59092971	3.30801573	4.40649045	1.43138595	1.35648628	1.96261782	1.82612132	1.07591756	1.45572127	1.23364535	1.75053825	1.19268948

16	17	18	19	20	21	22	23	24
H2O2	H2O2	H2O2	Isc	Isc	Isc	100% Prolifer:	100% Prolifer:	100% Proliferating Cells
33745747.7	47610763.7	52326504.7	46580990	48345274.3	51149997.8	31958614	45586275.5	51066727.5
1,806,750.67	2,645,268.33	4,379,011.00	3,255,154.33	4,042,458.33	5,819,002.00	1,512,222.67	2,379,445.00	1,179,888.00
5.35400989	5.55603004	8.36862891	6.98816047	8.36164112	11.3763485	4.73181555	5.21965213	2.31048289
0.56417386	0.58546154	0.88183656	0.73637097	0.88110022	1.19877223	0.4986107	0.55001603	0.243465
278,342.09	1,757,212.87	2,456,768.17	1,701,748.52	2,006,827.13	2,546,633.91	300,051.30	62,096.52	255,193.39
0.82482122	3.69078909	4.69507411	3.65331119	4.15103061	4.97875664	0.93887458	0.13621758	0.49972537
0.28451922	1.2731249	1.61954954	1.26019703	1.43188362	1.71740484	0.32386153	0.04698778	0.17237853
